# Progressive Accumulation of Activated ERK2 within Highly Stable ORF45-Containing Nuclear Complexes Promotes Lytic Gammaherpesvirus Infection

**DOI:** 10.1371/journal.ppat.1004066

**Published:** 2014-04-10

**Authors:** Evonne N. Woodson, Melissa S. Anderson, Matthew S. Loftus, Dean H. Kedes

**Affiliations:** 1 Myles H. Thaler Center for AIDS and Human Retrovirus Research, University of Virginia, Charlottesville, Virginia, United States of America; 2 Department of Microbiology, Immunology, and Cancer Biology, University of Virginia, Charlottesville, Virginia, United States of America; 3 Department of Internal Medicine, Division of Infectious Diseases and International Health, University of Virginia Health Systems, Charlottesville, Virginia, United States of America; Florida State University, United States of America

## Abstract

De novo infection with the gammaherpesvirus Rhesus monkey rhadinovirus (RRV), a close homolog of the human oncogenic pathogen, Kaposi's sarcoma-associated herpesvirus (KSHV), led to persistent activation of the MEK/ERK pathway and increasing nuclear accumulation of pERK2 complexed with the RRV protein, ORF45 (R45) and cellular RSK. We have previously shown that both lytic gene expression and virion production are dependent on the activation of ERK [1]. Using confocal microscopy, sequential pull-down assays and FRET analyses, we have demonstrated that pERK2-R45-RSK2 complexes were restricted to the nucleus but that the activated ERK retained its ability to phosphorylate nuclear substrates throughout infection. Furthermore, even with pharmacologic inhibition of MEK beginning at 48 h p.i., pERK2 but not pERK1, remained elevated for at least 10 h, showing first order decay and a half-life of nearly 3 hours. Transfection of rhesus fibroblasts with R45 alone also led to the accumulation of nuclear pERK2 and addition of exogenous RSK augmented this effect. However, knock down of RSK during bona fide RRV infection had little to no effect on pERK2 accumulation or virion production. The cytoplasmic pools of pERK showed no co-localization with either RSK or R45 but activation of pERK downstream targets in this compartment was evident throughout infection. Together, these observations suggest a model in which R45 interacts with pERK2 to promote its nuclear accumulation, thereby promoting lytic viral gene expression while also preserving persistent and robust activation of both nuclear and cytoplasmic ERK targets.

## Author Summary

In this study, we find that lytic RRV infection leads to selective and progressive accumulation of pERK2 within RRV ORF45 (R45)-containing nuclear complexes in infected cells. In these complexes, pERK2 decays with first order kinetics and a half-life of nearly 3 hours, suggesting a highly stable complex with a slow R45 off-rate, while pERK1 decays with a half-life of less than 30 minutes, consistent with its accessibility to cellular phosphatases. We further describe that despite the apparent sequestration of pERK2 within the R45 complexes, downstream activation of pERK nuclear substrates remains robust, promoting virion production. Using confocal microscopy and FRET analyses, we show that R45 closely interacts with both pERK2 and pRSK2 in the nucleus in heterodimeric or heterotrimeric complexes. Lastly, although we demonstrate that RSK ectopic overexpression augments the levels of pERK2 in 293 cells co-transfected with R45, its role in ERK2 activation and virion production during RRV infection is not essential, in apparent contrast to its requirement during KSHV infection.

## Introduction

The life cycle of all herpesviruses has two distinct phases: lytic and latent. During lytic infection, nearly the entire genome is undergoing active transcription. In contrast, during latency, gene expression is limited to a few genes and, in most species, miRNAs [2]–[4]. The gammaherpesvirus, Kaposi's sarcoma-associated herpesvirus (KSHV), the causative agent of three human malignancies, strongly favors latency following initial entry into a variety of target cells in culture [5]–[9]. Much of our current understanding ofthe lytic phase in KSHV derives from studies on the reactivation of the virus from latently infected primary effusion lymphoma (PEL) cell lines. In these patient derived cell lines, the addition of reactivating agents such as phorbol esters or HDAC inhibitors results in reactivation (productive infection) in less than 25% of the cells, making the study of lytic replication challenging. In contrast, the nonhuman primate homolog, Rhesus monkey rhadinovirus (RRV) that closely resembles KSHV in both sequence and genomic structural organization, displays a robust lytic infection following de novo infection of cultured rhesus fibroblasts (RhF) [10]. This allows the study of lytic infection and its regulationwithout the addition of exogenous inducing agents or overexpression of early components of the lytic cascade that can confound results.

To establish infection and promote lytic replication within the host, viruses often manipulate cellular processes and pathways that play essential roles in the viral life cycle. Earlier studies demonstrated that KSHV and RRV infections result in the activation of the mitogen activated protein kinase (MAPK) pathways, including the extracellular signal-regulated kinase (ERK) pathway [1], [11]–[14]. Further, these investigations showed that viral gene expression and replication is dependent upon this activation [1], [12], [14]. Typically, a wide range of external stimuli such as growth factors, cytokines, and osmotic stress activate the MAPK signaling pathways. Upon activation, the signals propagate downstream through a series of phosphorylation events and are eventually converted into biological responses. For the prototypical ERK pathway, mitogenic stimulation, including viral infection at the cell surface, leads to the active shuttling of activated ERK (phosphorylated ERK; pERK) into the nucleus where it further phosphorylates downstream substrates such as transcription factors involved in cell survival, cell cycle, and proliferation [15]–[17]. Nuclear phosphatases then remove the phosphate groups, leading to the regeneration of inactive ERK pools that shuttle back to the cytoplasm [17]–[19].

Several of the transcription factors activated by ERK are required for KSHV lytic gene expression. The promoter of the lytic switch gene, replication and transcriptional activator, RTA (encoded by ORF50), which is both necessary and sufficient for lytic gene expression, contains AP-1 responsive elements. AP-1, activating protein-1, is a family of transcription factors, which are heterodimers composed of Fos, Jun, and ATF subunits, several of which are ERK substrates [20]–[23]. Deletion of these elements results in a significant decrease in RTA activation [22]. Finally, and not surprisingly, blocking the activation of ERK and, thus, its downstream substrates, leads to a significant decrease in virus production, suggesting a critical role for the ERK pathway in immediate early viral gene expression and progression through the lytic cascade [14].

In KSHV infection, like other viral infections, investigators have demonstrated that ERK activation occurs in two stages. The first results from viral envelope glycoproteins engaging cellular receptors, while the second coincides with the expression of viral genes [13], [24]–[27]. This second phase for KSHV is a “pre-latent” stage marked by the transient expression of a limited repertoire of viral genes that eventually leads to the establishment of latency and a concurrent decrease in activated ERK levels [13]. In contrast, following de novo infection of RhF with RRV, pERK levels continued to rise during this second phase, reflecting the initiation of the classic cascade of lytic gene expression that results in robust virion production and release. These observations, coupled with earlier KSHV work demonstrating i) a correlation between the decline in pERK levels and the establishment of latency, and ii) blockade of ERK activation prevents lytic replication, suggest that pERK is necessary at various stages in the viral life cycle. Further, many of the agents used to induce lytic reactivation, such as TPA and sodium butyrate, induce ERK activation. Taken together, these data led us to postulate that this pathway is important in modulating the balance between the lytic and latent phases of gammaherpesvirus infection [13], [14], [20], [28], [29].

Since the second phase of ERK activation in KSHV and RRV infection is likely due to viral gene expression, it is plausible that a particular gene(s) could be responsible for this activation. Several investigators demonstrated that exogenous expression of specific lytic KSHV genes could lead to the activation of ERK. These include the viral G-protein coupled receptor (ORF74, [30], [31]) and ORF45 [24], [32]. In light of the KSHV work describing the mechanism by which K45 induces sustained ERK activation, we chose to determine if RRV ORF45 (R45) functions similarly in de novo infection. ORF45 is a multifunctional protein that is involved in various stages of the lytic cycle including modulation of the immune response and egress of newly formed virions [33], [34]. In addition, for both KSHV and RRV, it is a component of the viral tegument [35]–[37]. A recent study from the Zhu laboratory suggested that activation of ERK and RSK/p90 ribosomal S6 kinase during KSHV lytic reactivation reflected their protection from cellular phosphatases following the formation of a heterotrimeric complex with K45 [24].

In the present study, we demonstrated that RRV employs a similar mechanism of ERK activation following de novo infection and that pERK levels continued to rise throughout the lytic cycle. We also found that heterotrimeric complexes with R45, pERK2 and RSK2 arose with overexpression of R45 alone. Likewise, following de novo RRV infection, co-immunoprecipitation analysis and confocal microscopy revealed that R45 interacts with both ERK and RSK in infected cells. FRET analyses of infected cells confirmed that a subset of R45 was tightly associated with both pERK and pRSK within the nucleus. In contrast, though R45, pERK2 and RSK were also present in the cytoplasm, we found no evidence of their interaction in this compartment. Finally, we present data showing that infection led to the activation of nuclear and cytoplasmic pERK targets, suggesting that the pERK in R45 or R45/RSK complexes in the nucleus retained their kinase activity.

## Materials and Methods

### Ethics statement

No animal or human subjects were involved in this study.

### Cell culture

Telomerase-immortalized rhesus fibroblasts (RhF) were maintained in Dulbecco's modified Eagle's medium (DMEM; Gibco) supplemented with 10% fetal bovine serum (FBS; Gibco), 100 mg/liter sodium pyruvate, and 500 ng/mL puromycin, as described previously [35]. 293 cells were maintained in DMEM supplemented with 5–10% FBS.

### Virus stocks

For RRV stocks, confluent RhF in a T182 cm^2^ flask (approximately 2×10^7^ cells) were infected with RRV strain H26–95 at a multiplicity of infection (MOI) of 0.05 for 1 hour, followed by supplementation with an additional 100 mL complete media/flask. Supernatants were harvested 5 days post-infection (p.i.). Viral supernatants were cleared of cellular debris by low speed centrifugation and subsequently passed through a 0.45-µm filter. Virus was then pelleted by high-speed centrifugation for 3 hours at 12,855× g in a Sorvall SL250T rotor and the resulting pellet was resuspended overnight at 4°C in a final volume of 1.0 mL of TNE (20 mM Tris, pH 7.5, 100 mM NaCl, 1 mM EDTA).

### Reagents

#### Antibodies

Antibodies detecting total ERK1/2 (1∶750), phospho-MNK1 (1∶500), total MNK1 (1∶500), phospho-Elk-1 (1∶500), total Elk-1 (1∶500), total RSK2 (1∶100 for immunofluorescence staining; 1∶500 for western blot), phospho-RSK (T573; 1∶500 and, of note, this antibody was used throughout the study), total MEK (1∶750), and phospho-MEK (1∶750) were purchased from Cell Signaling Technology (tested by manufacturer to detect these proteins across species by western blot); total MNK1 (1∶200) for immunofluorescence microscopy from Novus Biologicals (tested by manufacturer to detect MNK1 by IF); pERK1/2 (1∶10,000 for immunoblotting and immunofluorescence; the epitope recognized by the antibody is in the regulatory site of active MAP kinase and is completely conserved in monkey), HA and FLAG (1∶500) antibodies from Sigma-Aldrich; total RSK1 from Upstate (1∶500); and RanBP (1∶7,500) antibodies from BD Biosciences; GAPDH (1∶500) from Santa Cruz. Anti-RRV Major Capsid Protein (MCP, 1∶200) was kindly provided by Scott Wong at Oregon Health and Science University. Anti-RRV ORF45 Ab polyclonal antibody (1∶7,500 for immunoblotting; 1∶250 for immunofluorescence staining) was raised in rabbits and purchased from Open Biosystems, Inc. RRV R45 antibody was also conjugated to Alexa-Fluor 594 and Alexa-Fluor 488 for immunofluorescence staining using a monoclonal antibody labeling kit (1∶125; Invitrogen). IRDye-800 anti-mouse, IRDye-800 anti-rabbit, and IRDye-680 anti-rabbit (all 1∶10,000) were purchased from LiCor Biosciences and Rockland Immunochemicals.

#### siRNAs

ON-TARGETplus SMARTpool siRSK1, siRSK2, and siGENOME Non-Targeting siRNA #5 (siRNA negative control) were purchased from Thermo-Scientific.

### Plasmid constructs

pKH3-RSK2 was provided by D. Lanigan (Vanderbilt University). pCMV-Tag3a was a gift from J. Lawrence (University of Virginia). An R45 PCR product from the RRV genome was cloned into the pCMV-Tag3a backbone, and a FLAG-tag was inserted at the N-terminus of the gene for immuno-detection.

### Transfections

For 293 transfections, cells were seeded into plates, incubated overnight at 37°C, and transfected with 3–4 ug total DNA in 6-well tissue culture plates in serum free conditions per the Lipofectamine 2000 protocol (Invitrogen). Four to six hours post-transfection, transfection media was removed and replaced with complete 293 complete media. Cells were harvested 48 hours post-transfection.

### Protein electrophoresis and immunoblot analysis

Cells were lysed with whole cell lysis buffer (50 mM Tris, pH 7.3, 150 mM NaCl, 1% Nonidet P-40, 5 mM EDTA, 10% glycerol) supplemented with 1 mM sodium orthovanadate (Na_3_VO_4_), 40 mM β-glycerophosphate, 30 mM sodium fluoride, 1 mM phenylmethylsulfonyl fluoride, and 1× protease inhibitor cocktail (Roche Applied Science) just prior to use. Cell lysates and virus samples were reduced in sample buffer (lithium dodecyl sulfate [LDS]) with reducing agent containing 0.5M dithiothreitol [NuPage; Invitrogen]), and proteins were separated by sodium dodecyl sulfate polyacrylamide gel electrophoresis (SDS-PAGE) on 10% Bis-Tris gels (Invitrogen).

For immunoblot analyses, proteins separated by SDS-PAGE were transferred to nitrocellulose membranes for 60 min at 250 mA at 4°C. The membranes were blocked in 5% nonfat milk-TBS (20 mM Tris base, 150 mM NaCl, 3 mM Tris-Cl) for 1 h at room temperature and then incubated with primary antibodies for 2 h at room temperature or overnight at 4°C. After three washes with TBS-Tween (0.05%) at room temperature, membranes were incubated with secondary antibodies (45 min, room temperature). For semi-quantitative enhanced chemiluminescence (ECL) immunoblotting, membranes bound to primary Abs were incubated with (1∶5,000–1∶10,000) horseradish peroxidase-conjugated secondary antibodies (Jackson ImmunoResearch), and Western Lightening chemiluminescent reagent (Perkin-Elmerrpar; was used according to manufacturer's protocol. For all quantitative immunoblotting, membranes were incubated with InfraredDye-800 conjugated anti-mouse or anti-rabbit and InfraredDye-680 conjugated anti-mouse diluted 1∶10,000 in TBS-Tween (0.05%). Images were scanned and analyzed using Odyssey Infrared Imaging System and 3.0 Software (LiCor Biosciences).

### Immunoprecipitation

Cell lysates (100–250 ug) were first pre-cleared by incubating lysates with TrueBlot Rabbit Ig beads (eBioscience) for 30–60 min at 4°C tumbling end-over-end. After the incubation, supernatants were collected after beads were pelleted at maximum speed in a microfuge for 5 min at 4°C. Beads and immunoprecipitating antibody (2 ug) were added to pre-cleared supernatant. For sequential immunoprecipitations, cell lysates were incubated with either EZview Red anti-Flag M2 or anti-HA affinity resin; Sigma-Aldrich. Samples were incubated overnight at 4°C tumbling end-over-end. Beads were washed 3–5 times in RIPA buffer (50 mM Tris-HCl pH 7.4; 1% NP-40; 0.25% sodium deoxycholate; 150 mM NaCl; 1 mM EDTA). After the last wash, the supernatant was aspirated and 50 uL of sample buffer was added directly to bead pellet nd samples were boiled prior to protein electrophoresis and immunoblot analysis (see above).

### siRNA

siRNA oligonucleotides were transfected into RhF using Lipofectamine RNAiMAX (Invitrogen) at a concentration of 10 nM as per manufacturer's protocol. Twenty-four hours post-transfection, cells were infected at MOI of 5 for 1 h at 37°C. Residual virus was removed by sequential PBS washes, and fresh media was added. Cells were harvested 48 h p.i.

### Infections for time course

For all conditions, 2.5×10^5^ cells were plated in 6-well plates in 2 mL complete media. Cells were incubated at 37°C overnight. Prior to infection, media was removed, and cells were washed once with 1× PBS. 1 mL of serum-free (SF) media was added to each well, and cells were placed on ice. Cells were incubated with or without virus (see below) for 1 h on ice while rocking.

#### For RRV-infected cells

Frozen stocks of RRV were thawed, and the appropriate volume (MOI 10) was added to wells containing SF media. For UV-RRV infected cells, inactivated RRV was prepared by incubating viral stocks with 10 mJ/cm2 UV light for 10 minutes as previously described [38], [39]. An equal volume of the UV-irradiated virus was added to each well to achieve an MOI of 10.

#### For uninfected (UI) cells

An equal volume of 1× PBS (based on the volume of virus used in UV-RRV and live-RRV conditions) was added to each well.

After infection (1 hr on ice), virus was removed with two PBS washes and the first time point (time zero) was collected. In all other wells, complete media was added and cells were incubated at 37°C until harvested at various time points post-infection (p.i.).

### Plaque assays

As described previously [40], 48 h after RhF were plated in 12-well plates, pre-cleared supernatant from each siRNA condition was serially diluted 5-fold and applied to cells for 1 h at 37°C. Each dilution was performed in duplicate. Overlay media containing methylcellulose (0.6%) was added to plates containing viral inoculum. Plates were incubated for 5 days at 37°C. Overlay media was then removed, and cells were stained with crystal violet (0.8%) for 10 minutes at room temperature. Plaques were counted under 10× magnification using an inverted microscope (Nikon Eclipse TE-2000-E). Absolute titers were determined based on number of plaques and dilution used.

### Statistical analysis

Quantitative data are presented as means and standard errors of the means (SEM). Statistically significant differences between conditions were determined using an unpaired Student's *t* test. All calculations were performed using GraphPad Prism online software. To denote statistical significance, we used the following *P* value conventions: *P*>0.05, not statistically significant (ns); *, *P*<0.05; **, *P*<0.01; and ***, *P*<0.001.

### Indirect immunofluorescence staining

Cells were seeded into 8-well chamber slides and infected with RRV. At 24 or 48 h p.i., cells were fixed with 4% formaldehyde in phosphate buffered saline (PBS) solution for 15 min at room temperature, permeabilized with 0.02% Triton X-100 (v/v) in 10% normal goat serum (NGS)/PBS for 20 min at room temperature, washed twice with 0.05% Tween-20/PBS (PBS-T), and incubated with primary antibody (1∶250 pERK, 1∶100 RSK2, 1∶125 Alexa Fluor R45 conjugates) for 1 h at room temperature or overnight at 4°C. After three PBST washes, secondary antibodies were diluted in 10%NGS/PBST and cells were incubated with secondary antibodies for 30 min at room temperature. After secondary antibody staining, cells were counterstained with DAPI (4,6-diamidino-2-phenylindole; Sigma) (0.5 ug/mL) for 4 min to detect nuclei. DAPI was omitted in FRET analyses. Coverslips were mounted on 8-well chamber slides with Fluoro-Gel (Electron Microscopy Sciences) and visualized by standard fluorescence microscopy or confocal microscopy.

### Phase-contrast microscopy

Images of RhF were obtained with a Nikon Eclipse TE-2000-E microscope and an ORCA-ER digital chargecoupled-device (CCD) camera (Hamamatsu) and saved as tiff files using Openlab 5 software (Improvision; Perkin Elmer).

### Confocal microscopy

Images of RhF were obtained using a Zeiss LSM 710 multiphoton confocal microscope. Each image was taken with a 63× objective lens with oil. Images were saved as tiffs from the Zeiss software and analyzed using Image J software.

### Fluorescence resonance energy transfer (FRET) microscopy imaging

For FRET microscopy, Alexa488 and Alexa555 served as the donor and acceptor fluorophores, respectively. The FRET efficiency (*E*) was quantified using two separate methods: the acceptor photobleaching FRET (APFRET) method and the processed FRET (PFRET; filter-based) method that is based on the detection of the acceptor sensitized emission. Both measurements were carried out on a Leica SP5 X confocal microscope imaging system, which was described earlier [41]. All images were acquired using a 60×/1.4NA oil-immersion objective lens. The Leica system carries a white light laser module tunable from 470 to 670 nm in 1-nm increments [41], from which the 488-nm and 555-nm laser lines were selected as the Alexa488 and Alexa555 excitation wavelengths, respectively. Laser powers are controlled through acousto-optical tunable filters and were optimized for each excitation wavelength. Two emission channels were set up using acousto-optical beamsplitter and two identical photomultiplier tubes: the 500–545 nm range for Alexa488 and the 565–650 nm range for Alexa555.

APFRET imaging was conducted on pERK (Alexa488)-R45 (Alexa555) samples. Two imaging channels were used in APFRET imaging: the donor channel uses the 488-nm excitation wavelength and measures signals in the 500–545 nm emission range; the acceptor channel employs the 555-nm excitation wavelength and captures signals in the 565–650 nm emission range. Images of both channels were acquired from the double-label specimens before and after photobleaching the acceptors in a selected region of interest (ROI). Identical imaging conditions were applied before and after photobleaching. The average intensity of the bleaching region is determined from the pre- and the post-bleach images: *IDA_pred_* and *IDA_prea_* are measured from the pre-bleach donor and acceptor channels, respectively; *IDA_postd_* and *IA_posta_* are measured from the post-bleach donor and acceptor channels, respectively. *IDA_prea_* and *IDA_posta_* were compared to make sure the photobleaching was sufficient when *IDA_posta_* should be less than 10% of *IDA_prea_*. Since only a small ROI is bleached at a time, the photobleaching process took only a few seconds. As a control, we also applied the same bleaching conditions to the donor-alone (Alexa488) samples and measured the donor intensity before (*ID_pred_*) and after (*ID_postd_*) photobleaching to ensure that the donor was not affected by the acceptor photobleaching process – the difference between *ID_pred_* and *ID_postd_* is negligible. The FRET efficiency (*E*) was calculated as: *E* = 1−(*IDA_pred_*/*IDA_postd_*), where *IDA_pred_* and *IDA_postd_* are the fluorescence intensities of the quenched donor and unquenched donor, respectively.

PFRET imaging was also conducted on both the R45 (Alexa555)-pERK (Alexa488) and R45 (Alexa488)-RSK2 (Alexa 555) samples. Three imaging channels were used in PFRET imaging: the donor channel (the 488-nm excitation wavelength and the 500–545 nm emission range) quantifies the quenched donor (qD) signal level; the FRET channel (the 488-nm excitation wavelength and the 565–650 nm emission range) detects the uncorrected FRET (uFRET) signal which contains the FRET signal (called PFRET here) as well as the bleedthrough contaminations from both the donor and the acceptor; the acceptor channel (the 555-nm excitation wavelength and the 565–650 nm emission range) measures the acceptor (A) signal level. To remove spectral bleedthrough, the single-label (pERK-Alexa488 and R45-Alexa555 or R45-Alexa488 and RSK2-Alexa555) control cells were also imaged at identical imaging conditions applied to the double-label cells. The signals of the acceptor-alone cells excited by the 555-nm laser line were checked in the donor (500–545 nm) emission range to make sure there is no back bleedthrough of the acceptor to the donor channel. Unlabeled cells were used to check the autofluorescence background level, which is negligible. The acquired double-label and single-label images were processed using the PFRET algorithm [42]–[44], which can accurately estimate and remove the spectral bleedthrough contaminations for different intensity levels, calculate the FRET signal, and quantify the FRET efficiency (E) at a pixel-by-pixel based level. The PFRET software also produces the results for selected regions of interest (ROIs) for quantitative FRET data analysis.

## Results

### Sustained ERK activation during de novo RRV infection

**Figure 1:**
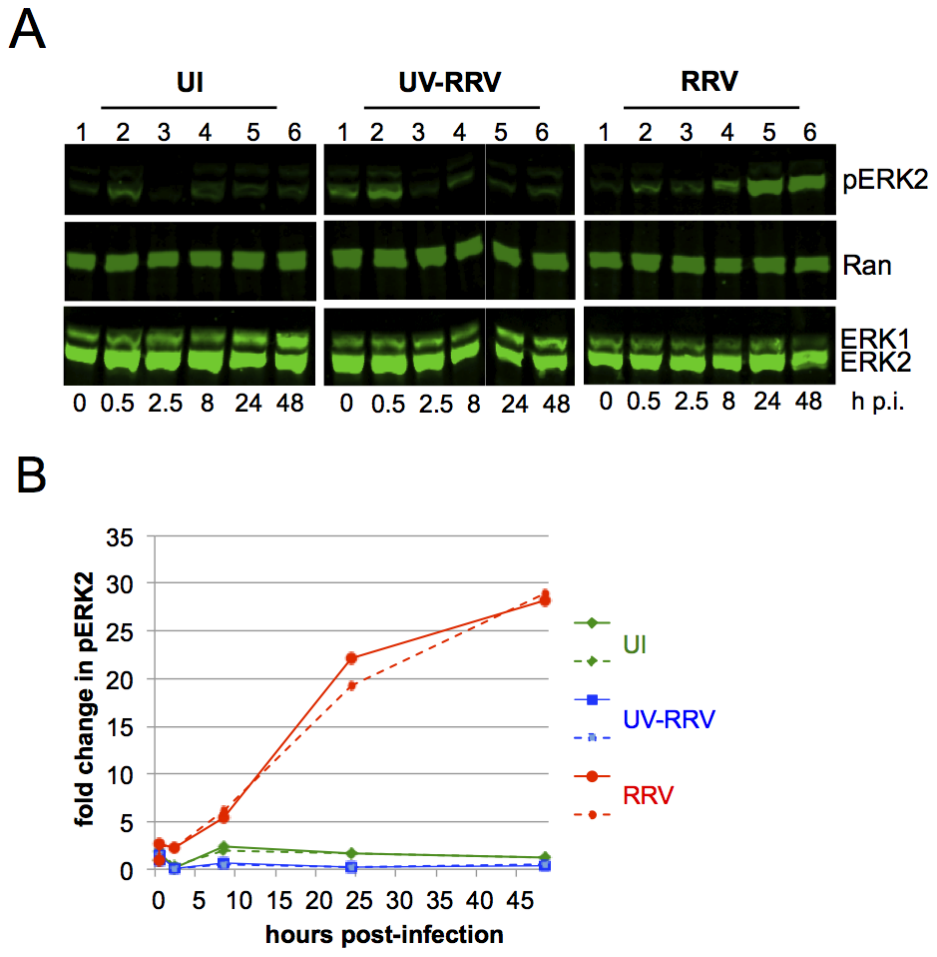
ERK activation is sustained in de novo RRV infection. (**A**) Quantitative immunoblots of uninfected (UI; left panels), UV irradiated-RRV infected (UV-RRV; MOI of 10 pre UV treatment; middle panels), or RRV-infected RhF (MOI of 10; right panels). 10 ug of cell lysate (10–15% of total lysate) was loaded per lane. Blots were probed with the following antibodies: anti-phosphorylated ERK (pERK), anti-Ran (loading control for cellular extracts), and anti-ERK1/2. (**B**) Graphical representation of the fold change in pERK2 over time relative to time zero. pERK2 values were normalized to Ran to correct for loading differences. Normalized pERK2 values were compared to time zero for each of the three conditions to determine the fold change. Two representative experiments are plotted; solid and dashed lines symbolize two separate experiments. Green, blue, and red lines represent UI, UV-RRV, and RRV respectively.

Our previous studies demonstrated that de novo RRV infection of rhesus fibroblasts (RhF) resulted in the activation of the MEK/ERK pathway and that this activation was necessary for early lytic gene expression and, thus, virus production [1]. Earlier work with primary KSHV infection of both human foreskin fibroblasts (HFF) and dermal microvascular endothelial cells (HMVEC-d) also demonstrated activation of ERK but, in those systems, levels of pERK fall beginning approximately 36 h post-infection, prior to the establishment of latency [13]. Although RRV can establish latency in rhesus monkey B cells [45], [46], we sought, instead, to determine the pattern of ERK activation in the purely lytic model system involving RRV infection of RhF. We predicted that a robust lytic infection would be marked by an even more prolonged, rather than transient, rise in activated ERK. We also asked how the mechanism of ERK activation compared to that evident with KSHV infection and reactivation. We infected RhF with either UV-irradiated (replication-incompetent) or replication competent RRV and measured the levels of pERK2 at increasing times post-infection (p.i.) by quantitative immunoblotting (Figure 1A). Since we showed previously that RRV infection preferentially activated ERK2, we quantified the changes in pERK2. For controls, we measured, in parallel, the pERK2 levels in uninfected (UI) cells.

In the UI cells (Figure 1A, left panel), the change of media alone led to a transient small (<2-fold) increase in the levels of pERK within the first 30 minutes. At 2.5 h p.i., pERK2 levels returned to baseline and remained low through 48 h. In RhF exposed to UV-irradiated (replication-incompetent) RRV (Figure 1A, middle panel), the levels of pERK2 also increased ∼2-fold within 30 minutes, and the levels at later time points were statistically indistinguishable from those in UI cells. Finally, infection of RhF with replication-competent RRV similarly led to pERK2 levels that rose ∼2-fold over baseline. In contrast to UI and UV-RRV conditions, however, the decline in pERK2 levels in RRV infected cells between 30 minutes and 2.5 hours (Figure 1A, right panel, lanes 2 and 3) was less pronounced (15% compared to 87% in UI and 97% in UV-RRV). Most remarkably, beginning at 8 h p.i., pERK2 levels increased markedly, rising 28-fold over baseline by 48 h. Of note, pERK2 levels continued to increase through 72 h p.i. but cell lysis at that time point made consistent cell collection difficult (data not shown). Figure 1B shows a graphical representation of the data from two independent and representative time course experiments of these same conditions. Taken together, these results demonstrated that unlike its human homolog, KSHV, which downregulates ERK activation as it assumes latency p.i. in culture, the lytic-prone RRV induces ongoing ERK activation in RhF following de novo infection.

### Activated ERK remains nuclear throughout RRV infection

**Figure 2:**
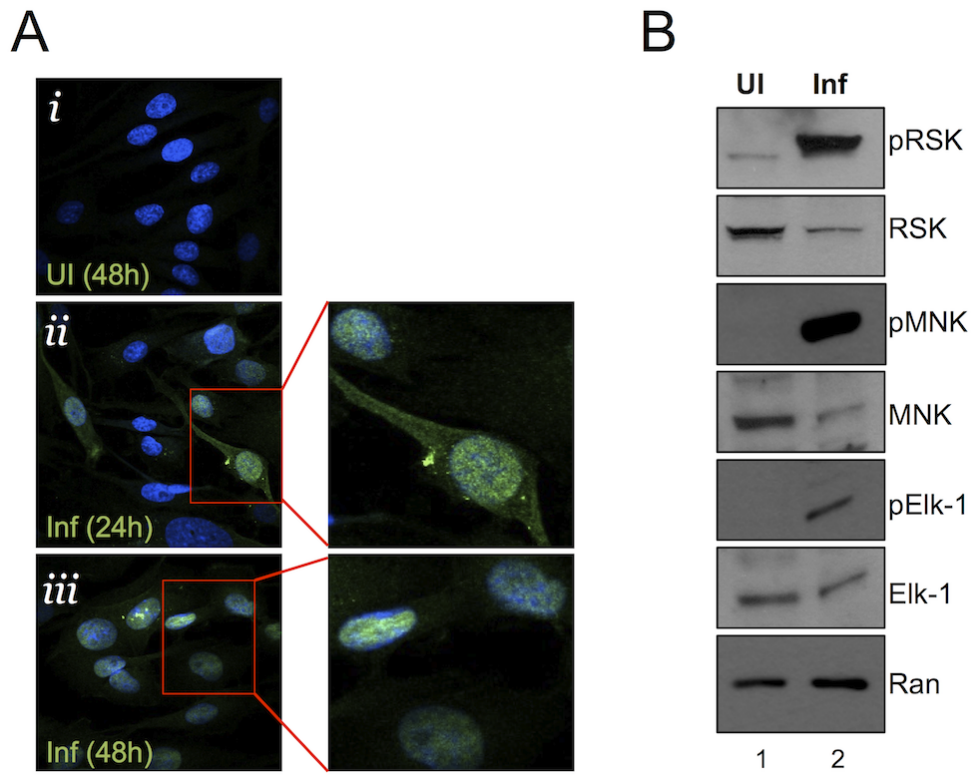
Nuclear localization of pERK with persistent phosphorylation of nuclear and cytoplasmic ERK targets. (**A**) Immunofluorescence images of UI (*i*) and infected (MOI of 5) (*ii, iii*) RhF. Cells were fixed, permeabilized, and co-stained with DAPI (blue) and pERK (red) and the two images were merged. Enlarged images from *ii* and *iii* are shown to the right of these images. (**B**) Immunoblot analysis of UI (lane 1) or infected (lane 2, MOI of 10) RhF collected 48 h p.i. Blots were probed with both phospho-specific and total antibodies for: RSK1/2 (top panels), MNK-1 (middle panels), Elk-1 (3^rd^ panels), and Ran to normalize for loading differences.

Since factors such as duration and magnitude of ERK activation can affect biological responses to extracellular stimuli [47], we asked whether sustained elevations in the levels of pERK2 from de novo RRV infection affected its localization and ability to target downstream substrates. We first examined the subcellular localization of pERK by staining for pERK in cells at either 24 or 48 hours p.i. In UI cells we did not detect pERK (Figure 2A, i) but by 24 h p.i., we detected pERK in both the cytoplasm and the nucleus, though the signal in the nucleus was more intense (Figure 2A, ii). By 48 h p.i., nearly all the pERK in infected cells localized to the nucleus (Figure 2A, iii), consistent with previous descriptions of its nuclear translocation in response to a variety of non-viral stimuli in various cells lines [16], [47]–[49] and in RhF following 12-O-tetradecanoylphorbol-13-acetate (TPA) treatment (Figure S1).

### pERK retains its ability to activate cellular substrates late in RRV infection

ERK is almost exclusively cytoplasmic in quiescent cells; however, activated ERK (pERK) is normally able to shuttle between the cytoplasm and nucleus to phosphorylate its cytoplasmic and nuclear substrates [17], [47], [48]. Nevertheless, nuclear accumulation of pERK is usually transient (varying from a few minutes to several hours) in virally uninfected cells due to the activity of phosphatases, some of which are nuclear, targeting pERK [16], [17], [50]. With pERK increasingly concentrated in the nucleus p.i. with RRV, we hypothesized that ERK's downstream targeting could be skewed towards its nuclear over its cytoplasmic substrates. Alternatively, if aberrantly retained in a viral protein complex [24], pERK might be sequestered in an inactive form, unable to access potential substrates [51]. To test these possibilities, we immunoblotted RRV-infected whole cell lysates for both total and phosphorylated ERK substrates: two cytoplasmic targets, p90 ribosomal S6 kinase (p90 RSK, which is involved in translational regulation [52]–[54] and MNK1 (a MAPK interacting protein) acts as an eIF-4E kinase downstream of ERK [55], [56], and interestingly, its activation has been shown to be important for herpes simplex virus-1 (HSV-1) protein synthesis and replication [57], and Elk-1 (a nuclear transcription factor and the first identified ERK substrate [16], [17], [58] (Figure 2B). We used phospho-antibodies that detect ERK-specific phospho-sites, whereby an increase in the levels of these phosphorylated proteins would be attributed to ERK kinase, indicative of its activity in infected cells. Although the infected cells (Figure 2B, lane 2) contained slightly greater amounts of protein, as evidenced by the intensity of the Ran signal, the loading control, the total levels of RSK, MNK, and Elk-1 were lower than in uninfected cells (Figure 2B, lane 1), possibly due to virally mediated host shut-off [59], [60]. Despite their lower total levels, the phosphorylated forms of the cytoplasmic and nuclear substrates were markedly greater in infected cells at 48 h p.i than in uninfected controls. These data suggested that although pERK appeared to localize to and increasingly accumulate in the nucleus at 48 h p.i., activated ERK retained the capacity to activate both cytoplasmic and nuclear effectors.

Consistent with these results, confocal fluorescence microscopy demonstrated that RRV infection led to i) a marked increase in the amount and a shift to nuclear localization of activated RSK2 [52], [54]; ii) nuclear localization of activated Elk-1 [16], [17], [58], but with the formation of a punctate pattern superimposed on a more diffuse nuclear distribution; and iii) a shift from a diffusely cytoplasmic MNK1 to a perinuclear distribution, as others have reported following TPA activation of this MEK/ERK pathway (Figure S2) [56]. Together, these data indicate that at least a portion of the accumulating pERK was neither sterically hindered nor irreversibly sequestered in the nucleus and that it retained the capacity to phosphorylate its usual substrates from both cellular compartments.

### RRV ORF45 (R45) is necessary for prolonged ERK activation

The work of others suggests that the second (post viral binding) phase of ERK activation in de novo gammaherpesvirus infection is likely due to the expression of lytic viral proteins [13], [14]. Furthermore, Kuang et al. found that KSHV ORF45 (K45) though not possessing kinase activity itself, leads to the formation of complexes with ERK and RSK and protects the phosphorylated forms of both kinases from cellular phosphatases. Thus, by blocking dephosphorylation, K45 allows accumulation of pERK and its substrate pRSK as opposed to quickly returning to basal levels within minutes of stimulation [47]. Although de novo KSHV infection results in a rise in pERK through 20 h p.i. in 293 cells and 36 h p.i. in primary cells [13], [24], [32]), these levels return towards baseline as the infection becomes latent. In contrast, de novo RRV infection of RhF is purely lytic and, in parallel, we have found that it is marked by an even more pronounced and continual rise in pERK accumulation (Figure 1A). Furthermore, we found that R45 expression rose steeply between 8 and 24 h p.i. and even higher 48 h p.i., paralleling the rise in pERK at these times (compare Figure 1 and Figure S3A). Conversely, siRNA knockdown of R45 expression diminished pERK2 levels by over 60% 48 h p.i. (Figure S3B and C).

**Figure 3:**
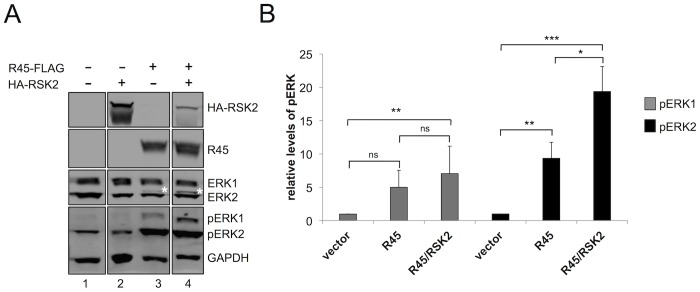
RRV ORF45 (R45) expression leads to the phosphorylation of ERK. (**A**) Immunoblots of 293 cells transfected with empty vector (lane 1), HA-RSK2 (lane 2), FLAG-R45 (lane 3), or co-transfected with FLAG-R45 and HA–RSK2 (lane 4) expression vectors. Cells were collected 48 h post-transfection and proteins from whole cells lysates were analyzed by quantitative immunoblotting (10–15% of total lysate was loaded per lane). Blots were probed with antibodies to detect the indicated proteins. Asterisk (*) above ERK2 represents pERK2. (The original blots from which this figure was derived is shown in Figure S8.) (**B**) Graphical representation of the fold change in pERK levels in 293 cells transfected with either vector alone (lane 1), FLAG-R45 (lane 2), or FLAG-R45/HA-RSK2 (lane 3). Data are representative of 3 independent experiments for pERK1 and 6 independent experiments for pERK2. P values were determined using Student's t test comparing the experimental condition to the control values. Asterisks above the bars denote the level of statistical significance. In comparing pERK1 levels from vector to R45 alone or to R45/RSK2, p values were 0.2005 and 0.0059 respectively; in comparing pERK1 levels from R45 to R45/RSK2, p value was 0.2264. In comparing pERK2 levels from vector to R45 alone or to R45/RSK2, p values were 0.0059 and 0.0006 respectively; in comparing pERK2 levels from R45 to R45/RSK2, p value was 0.0481.

To determine if RRV utilizes a conserved R45-dependent mechanism to promote its even more prolonged pattern of ERK activation and accumulation, we next measured pERK levels in 293 cells transfected with R45, RSK2, both or their respective empty vectors (Figure 3A). Endogenous ERK1 and ERK2 were present in cells transfected with empty vector and, though pERK2 levels were greater than pERK1 levels, both were relatively low. However, in cells transfected with R45 alone, but not RSK2 alone, levels of pERK2 rose by ∼9.4-fold compared to those in the control cells. Dual transfection with R45 and RSK2 augmented this rise in pERK2. Even though pERK1 levels also appeared somewhat higher in the presence of R45, this increase, compared to controls, did not reach statistical significance after repeated experiments. However, an increase in pERK1 occurred following exogenous expression of both R45 and RSK2. Figure 3B depicts graphically the quantification of three repetitions of the data in Figure 3A.

Although the expression of R45 alone led to an increase in pERK2 levels, Kuang et al. showed that pERK only remained phosphorylated in the presence of both K45 and RSK1/2, suggesting that all components of the complex in the case of KSHV infection must be present to protect these phosphoproteins from phosphatases [24]. To assess whether pERK2 accumulation during RRV infection also had this multi-component requirement, we co-expressed R45 and RSK2 and asked if the expression of both components would promote higher levels of pERK compared to R45 alone. As a control, we first transfected cells with RSK2 alone and found that pERK levels were not statistically different from those transfected with vector control (Figure 3A, compare lanes 1 and 3). In contrast, pERK2 levels were approximately 2-fold greater in cells transfected with both RSK2 and R45 compared to R45 alone, suggesting that an analogous complex may function to promote pERK accumulation in RRV. Additionally, we note the appearance of a slower migrating ERK2 band in lane 4 (denoted by *), which is indicative of an increase in pERK2 in the presence of RSK2 [1]. Levels of pERK1 appeared to be modestly higher in cells co-transfected with both RSK2 and R45 compared to those with R45 alone but these differences did not reach statistical significance; however, when compared to vector alone, these levels were significantly increased (Figure 3B). We obtained similar results in cells co-transfected with R45 and RSK1 (data not shown).

### R45 forms a complex with ERK and RSK during de novo RRV infection

**Figure 4:**
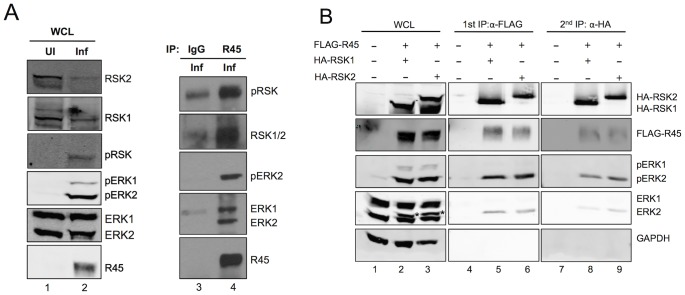
R45 forms a complex with ERK and RSK. (**A**) Immunoblots of RhF uninfected (lane 1) or infected with RRV (lane 2; MOI of 10) for 48 h. Blots were probed with the indicated antibodies. Whole cells lysates (10–15% of total lysate was loaded per lane) from lane 2 were immunoprecipitated with either rabbit IgG as a negative control (lane 3) or anti-R45 (lane 4). The immunoprecipitates were blotted with the antibodies indicated. (**B**) Immunoblots of 293 cells transfected with empty vector alone (lane 1) or co-transfected with FLAG-R45 and HA-RSK1 (lane 2) or HA-RSK2 (lane 3) and harvested 48 h after transfection. Cell lysates were immunoprecipitated with anti-FLAG affinity gel and eluted with 3× FLAG peptide (10–15% of total lysate was loaded per lane). The eluates were then sequentially immunoprecipitated with anti-HA affinity gel. Whole cell lysates and immunoprecipitates were analyzed by immunoblotting and probed with the antibodies indicated to the right of the blots. Starred bands (*) in lanes 2 and 3 represent pERK2.

To determine if an R45-RSK-ERK complex forms in RRV-infected cells as it does following KSHV reactivation, we immunoprecipitated the lysates from infected cells with an anti-R45 antibody, and probed for pRSK (see methods), RSK1/2, pERK, ERK1/2, and R45, using the antibodies indicated in Figure 4A (lanes 1, 2). Although low levels of total ERK, total RSK, and pRSK were present in the IgG pulldown control, the levels of ERK (and pERK) and RSK (and pRSK) were greatly enriched after the R45 IP (Figure 4A, lanes 3,4), suggesting that R45 associates with RSK and ERK in infected cells. Here we noted that although similar levels of total ERK1 and ERK2 associated with R45 after the R45 IP, we only detected pERK2 and not pERK1, the dominant phosphorylated isoform during RRV infection of RhF (Figure 4A, lane 4) [1]. Taken together these data suggested that R45 was capable of interacting with RSK1/2 and both isoforms of ERK, though may have preferentially associated with pERK2.

Although these data demonstrated that R45 interacted with ERK and RSK, they did not distinguish between two potential and distinct heterodimeric protein complexes that R45 could form separately (i.e. R45-pERK2 or R45-pRSK) and a three-protein complex containing R45, pERK2 and pRSK. To address this issue, we tested directly for the presence of all three components in a single complex, taking advantage of the ability to overexpress tagged versions of R45 and RSK within 293 cells followed by sequential immunoprecipitations (Figure 4B). Figure 4B, lanes 1–3, shows the total levels of input proteins that we detected using the antibodies indicated to the right of the figure. We first immunoprecipitated with anti-FLAG beads to pull down FLAG-R45 and then eluted with FLAG peptide. As we anticipated, RSK (pRSK) and ERK (pERK) immunoprecipitated with FLAG-R45 and were absent in cells transfected with empty vector (Figure 4B, lane 4–6). We noted that ERK2 was a doublet and the top bands (indicated by asterisks) represent the phosphorylated/active form of ERK2 that we have extensively characterized previously [1]. These data confirmed earlier experiments (see Figure 3), showing that R45 expression promoted the activation of ERK2. We then immunoprecipitated the eluates from the FLAG-R45 IP with anti-HA beads to pull down HA-RSK. FLAG-R45, ERK2, and pERK2 immunoprecipitated with the HA-RSK IPs but not in cells transfected with vector alone (Figure 4B, lanes 7–9). We obtained similar results whether we co-expressed HA-RSK1 or HA-RSK2. These data demonstrated that R45 could interact with both pERK and RSK in heterotrimeric complexes, similar to the KSHV model. Nevertheless, these experiments did not rule out the possibility of the concurrent existence of heterodimeric complexes representing the binary combinations of these three components.

### R45-containing complexes accumulate within the nuclei of transfected and infected cells

**Figure 5:**
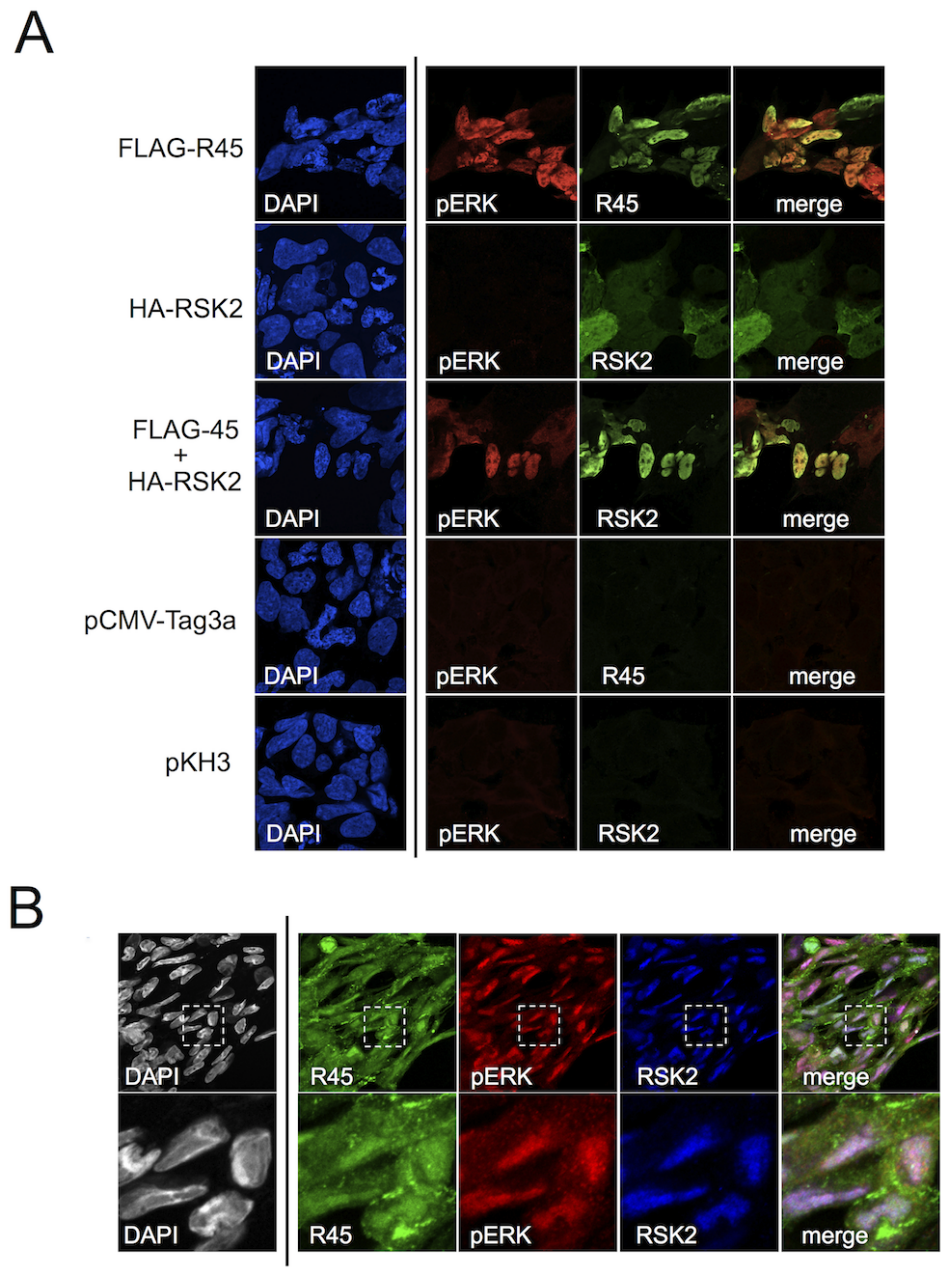
R45 interacts with pERK and RSK2 during de novo RRV infection. (**A**) Images of 293 cells transfected with FLAG-R45 (top row), HA-RSK2 alone (2nd row), or FLAG-R45 and HA-RSK2 (3^rd^ row). As controls, cells were transfected with either an empty FLAG vector (pCMV-Tag3a; 4^th^ row) or empty HA vector (pKH3; bottom row). The images in the 4^th^ column represent a merge of columns 2 and 3. (**B**) RhF infected with RRV (MOI of 5) for 48 h were fixed, permeabilized, and stained with the indicated antibodies, R45, pERK, and RSK2. The inset in each single stained image, denoted by a white box, was merged (columns 2–4 only) to produce the overlay (right-most panel). An enlarged image (4×) of the merged boxed area is shown below. Original magnification of 63×.

To corroborate the above IP data and to establish the intracellular distribution of the heterotrimeric (R45-RSK-pERK) complexes, we employed fluorescence microscopy on 293 cells expressing R45 with or without RSK2 48 h post-transfection. Although the relative levels of R45 and pERK did not always match perfectly, we found that R45 transfection led to pERK and R45 expression that generally co-localized within the nucleus (Figure 5A, top row), whereas HA-RSK2 transfection in the absence of R45 transfection resulted in both nuclear and cytoplasmic total RSK2 staining but no discernable pERK signal (Figure 5A, second row). In contrast, following R45 and HA-RSK2 co-transfection, RSK2 translocated to the nucleus and co-localized with elevated levels of pERK (Figure 5A, third row). Taken together, these imaging data were consistent with the pull down results that demonstrated the existence of the heterotrimeric complexes in transfected cells.

In light of our data demonstrating that the majority of pERK was in the nuclei of infected cells as late as 48 h p.i. (Figure 2A), we expected to detect RSK2 and R45 in the nucleus if the heterotrimeric complexes also formed during infection. To test this, we stained infected RhF with anti-R45, anti-pERK and anti-RSK2 (Figure 5B). As we anticipated, pERK, RSK2, and R45 were all present in the nucleus at 48 h p.i. [54]. In addition, we noted that although the relative levels of each protein varied, many nuclei demonstrated co-localization of all three components (Figure 5B, last column). The lack of perfect correlation between R45 and pERK could reflect the multifunctional nature of R45 and its cytoplasmic and nuclear subcellular localization. Nevertheless, taken together, these imaging data were consistent with the IP data, providing evidence for the formation of the R45-pERK2-pRSK2 trimeric complex within the nucleus during RRV infection.

**Figure 6:**
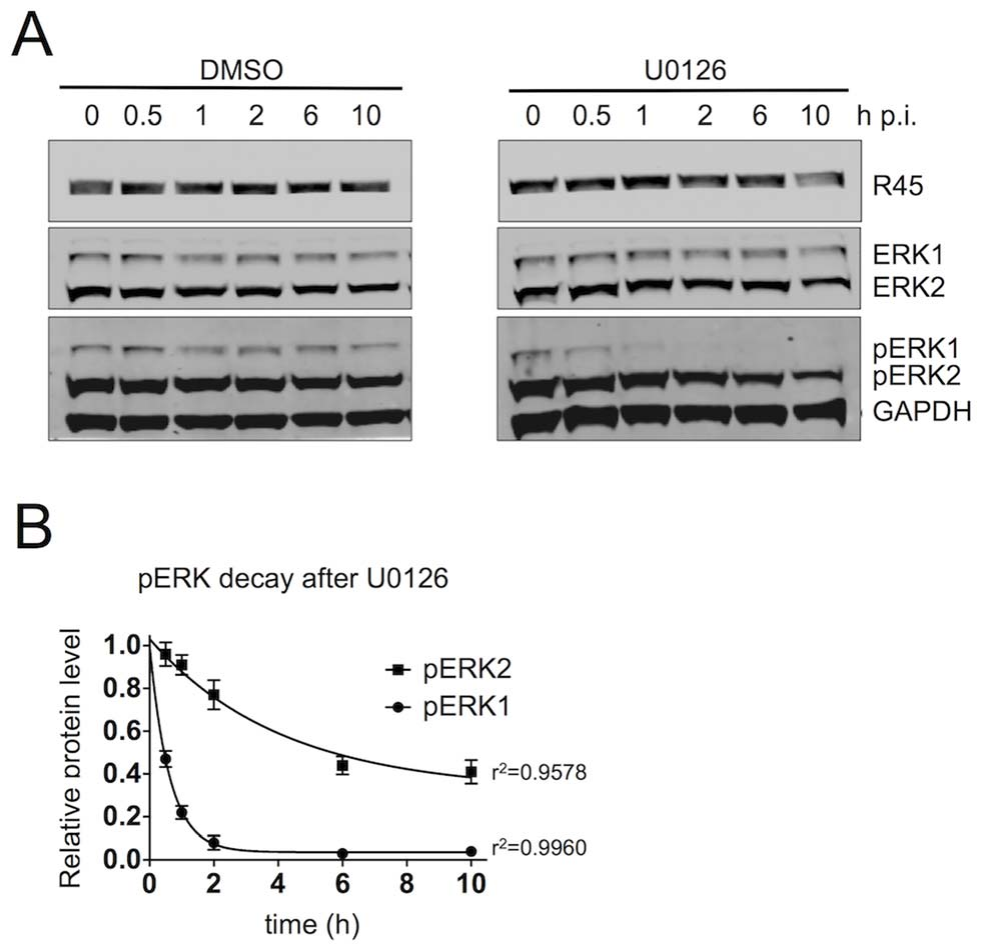
The R45-containing complex predominantly protects pERK2 over pERK1. (**A**) Quantitative immunoblots of RRV-infected RhF (48 h; MOI of 2.5) treated with DMSO (left) or the MEK inhibitor, U0126 (50 uM, [right]). 10–15% of total lysate was loaded per lane. Blots were probed with antibodies to phosphorylated ERK (pERK) and GAPDH. Times indicated above blots represents hours after U0126 treatment. (**B**) Graphical representation of decay of GAPDH normalized pERK1 and pERK2 levels following the addition of DMSO or U0126. Data are the mean of three independent experiments with error bars reflecting the SEM. R^2^ values indicate the coefficient of determination for each 1^st^ order exponential decay curve. (Of note, the lower MOI of 2.5 helped minimize the degree of lysis between p.i. hours 48 to 58).

To further characterize the nature of this complex, we sought to assess its stability during infection. Previous data from Zhu's laboratory suggest that when K45 is bound to pRSK, the interaction between pRSK and pERK is prolonged leading to the formation of a more stable trimeric complex [24]. To assess the stability of the R45 nucleated trimeric complexes in even greater detail, we infected RhF for 48 h, allowing the accumulation of complex-protected pERK, and then determined the half-lives of both pERK2 and pERK1 by measuring their levels following the addition of the MEK inhibitor U0126 or DMSO (Figure 6A). This drug would block the formation of additional pERK1 and pERK2. Of note, we previously showed that U0126 treatment prior to infection greatly reduced the levels of pERK1 and 2, R45, and other viral proteins, and thus virus production [1]. We hypothesized that stable and phosphatase-resistant complexes would lead to persistent elevations of pERK2 levels even without ongoing MEK activity. Following the addition of U0126 48 h p.i., pERK2 levels remained elevated with a prolonged t_1/2_ of approximately 3 h, likely reflecting a slow off rate of R45 from the complex and corroborating our hypothesis (Figure 6B). Of note, the GAPDH normalized R45 levels declined only minimally over the 10 h and were not statistically different between the DMSO and U0126 treated samples. In marked contrast, the levels of pERK1 decreased rapidly with, a t_1/2_ of approximately 25 min (Figure 6B). These latter data suggested that, in infected cells, pERK1, in contrast to pERK2, was freely accessibly to the enzymatic activity of MAP kinase phosphatases (MKPs) [18], [61], [62]. Of note, U0124, the inactive analog of U0126 had no effect on pERK accumulation during RRV infection (Figure S4). Together, these data argued that the R45-based complexes were highly stable and predominantly protected pERK2 rather than pERK1 from MKPs.

### FRET analysis supports direct interactions of R45 with pERK and RSK2 within the nuclei but not the cytoplasm of infected rhesus fibroblasts

**Figure 7:**
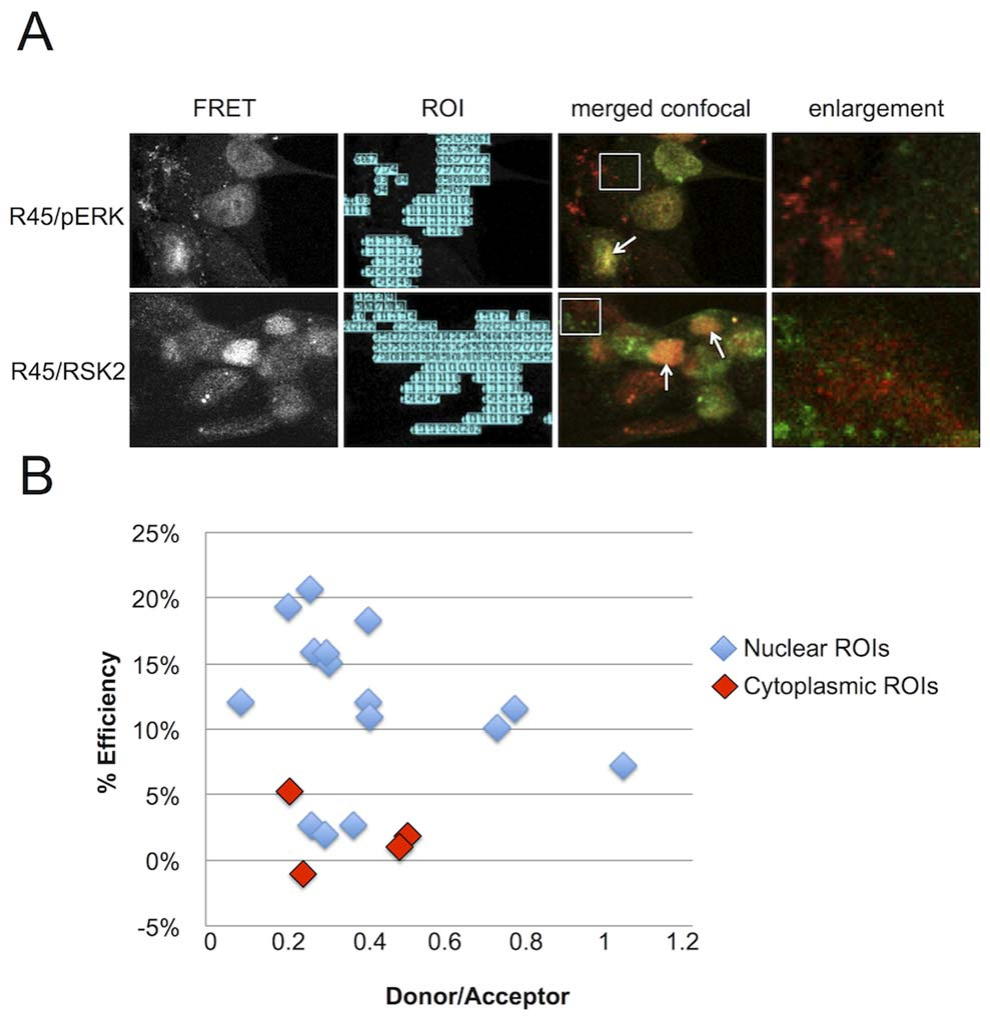
FRET analysis of RRV infected RhF reveals R45 in close proximity to both pERK2 and pRSK2 within the nuclei, but not the cytoplasm. (**A**) 1st column: Representative uncorrected (without background subtraction) FRET images of RRV-infected RhF stained with (top) anti-pERK (AF 488, donor) and anti-R45 (AF 555, acceptor) or (bottom) anti-R45 (AF488, donor) and anti-RSK2 (AF 555, acceptor). 2^nd^ column: software-generated regions of interest/ROIs (from 1st column) used for precise FRET (PFRET) analysis (see below). 3^rd^ column: Merged confocal images from the same fields as in first two columns. 4^th^ column: Enlarged images of the cytoplasm (4.4× magnification) from boxed region in 3^rd^ column. Arrows indicate examples of nuclei where a FRET signal is present and proteins co-localize by standard confocal microscopy. (**B**) Acceptor photobleaching (AP) FRET from RRV infected cells (MOI of 5). The acceptor was bleached for each region of interest (ROI; see also (A) above, column 2) within the nuclei (blue diamonds) and cytoplasm (red diamonds) to determine the efficiency of energy transfer. Efficiency of energy transfer was calculated for each point using the following equation: E% = (Donor post bleach - Donor prebleach)/Donor post bleach. E% less than 5% was discounted as background.

To more rigorously assess the proximity of the interaction between R45 and pERK or RSK2, we subjected infected cells to fluorescence resonance energy transfer (FRET) microscopy imaging (Figure 7). Focusing our attention on regions of co-localization, we used filter-based FRET (PFRET, see Methods) to measure of the efficiency of energy transfer from donor (Alexa Fluor 488, AF 488) to acceptor (Alexa Fluor 555, AF 555) fluorophore. PFRET is a quantitative intensity-based approach that analyzes traditional FRET data (see Methods) [63], [64]. The software can automatically generate regions of interest (ROIs) where a FRET signal is detected within the sample or based on user-defined parameters (Figure 7A, left-most panels). We created 10×10 pixel ROIs in both the nucleus and cytoplasm of RRV-infected cells; however, due to the intensity of the FRET signal in the nucleus, the majority of the ROIs were drawn in this cellular compartment (Figure 7A, 2^nd^ column). We noted efficient energy transfer from AF 488 (donor) to AF 555 (acceptor) (FRET efficiency ranging from 23 to 27%; Table S1). Since FRET efficiency (E%), which represents the proportion of donor molecules that have transferred excitation state energy to the acceptor molecules, is inversely related to the intermolecular distance (r) between the fluorophores used to label the antibodies (R45, pERK, and RSK2), we hypothesized that AF 488 and AF 555 would be further apart (e.g. whereas FRET allows resolution down to 1–10 nm, we postulated r to be 5–10 nm versus 1–5 nm) [63]. For FRET pairs pERK-AF 488/R45-AF 555 and R45-AF 488/RSK2-AF 555 within nuclei, r was 4.5 and 4.7, respectively [65], [66], though there were a few cells in which we did not detect any interaction, possibly representing cells at earlier stages of lytic infection with low intensity R45 staining. No significant FRET signal was evident within the cytoplasm of these two pairs even though we detected each component separately (Figure 7A, fourth columns). To confirm these results, we also performed acceptor photobleaching (AP) FRET on a separate R45-pERK sample (15 nuclei and the matched cytoplasm from 4 of the cells). This method also showed that the efficiency of energy transfer between pERK and R45 was markedly greater in the nuclei than in the cytoplasm of infected cells (Figure 7B). Together, these data corroborated the immunoprecipitation and microscopy data, suggesting that R45 forms a close interaction with pERK2 and RSK2 only within the nuclei of infected cells.

### Sustained ERK activation persists even in the near absence of pRSK

**Figure 8:**
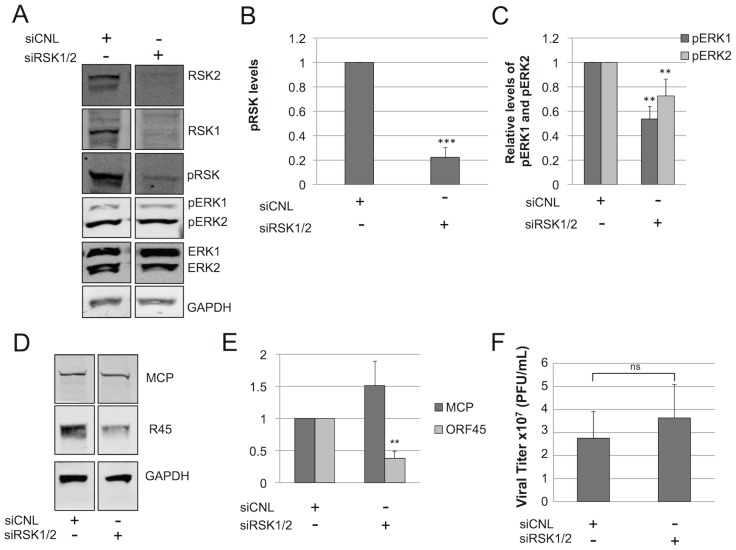
RSK is not required for ERK activation. Immunoblots of RhF transfected with non-targeting siRNA (siCNL) or sRSK1+siRSK2, as indicated. (**A**) 24 h post-transfection cells were infected with RRV (MOI of 5) and then harvested at 48 h p.i. Cell lysates were probed with the indicated antibodies (10–15% of total lysate was loaded per lane). The quantitative immunoblot shown is representative of three separate experiments. (Of note, the original blot from which these two lanes were derived is shown in Figure S9.) Graphical representation of the expression of (**B**) pRSK (pRSK2, T573 antibody); compared to siCNL, pRSK levels in the siRSK condition were statistically different; p = 0.0005 or (**C**) pERK isoforms treated with siRSK1 plus siRSK2 or siRNA control; compared to siCNL, pERK1 and pERK2 levels in the siRSK condition were statistically different (p = 0.0024 and 0.0014. respectively). (**D**) Quantitative immunoblot of whole cell lysates from a separate siRSK experiment was probed with antibodies to two structural viral proteins, MCP (top panel) and R45 (middle panel) and normalized to GAPDH (bottom panel) to correct for loading (10–15% of total lysate was loaded per lane). (**E**) Graphical representation of (D); compared to siCNL, R45, but not MCP, levels in the siRSK condition were statistically different (p = 0.0042 and 0.38, respectively). (**F**) Viral titers in the supernatants from cells in (A); compared to siCNL, titers from supernatants in the siRSK condition were not statistically different (p = 0.50). For (A) through (F), data represent the mean +/− SEM of 3 separate experiments. P values were determined using Student's t test comparing the experimental and control conditions. Columns lacking asterisks were statistically indistinguishable from corresponding siCNL values.

For KSHV reactivation, Zhu and colleagues reported that pRSK acts as a bridge between K45 and pERK, suggesting that it is required for sustained ERK activation. Our data also pointed to the existence of the homologous heterotrimeric complex containing all three of these components in the RRV system and that RSK augmented R45 in elevating pERK levels in transfected cells. To determine if pERK accumulation during RRV infection was also dependent on pRSK, we targeted both RSK1 and RSK2 with siRNA (Figure 8A). With an RSK1/2 knockdown between 70–80%, pRSK levels dropped concomitantly (∼80%) by 48 h p.i. compared to levels in siCNL treated cells (Figure 8B). Despite this level of pRSK/RSK knockdown, pERK2 accumulation persisted and, by 48 h p.i., maximal levels were decreased by only ∼25% compared to controls (Figure 8C).

If pRSK played a non-essential role in maintenance of pERK levels during RRV infection, we also predicted that the pERK half-life following the addition of the MEK inhibitor U0126, as we had measured previously (Figure 6), would be similar in RRV infected cells following pre-treatment with either control or RSK-directed siRNA. To test this prediction, we infected RhF 24 hours after siRNA reverse-transfection and then waited 48 h p.i. before adding U0126 and measuring pERK 1 and 2 over the subsequent 10 hours, as we have described above. We found that the half-lives of pERK2 in infected cells pre-treated with siRNA to RSK or siRNA control were statistically indistinguishable and were prolonged (t_1/2_ = 1.34 and 1.23 hours, respectively). For pERK1, siRSK pre-treatment actually appeared to lead to a modest increase in half-life compared to siCNL (t_1/2_ = 40 vs. 11 minutes, respectively) but both decay curves were statistically distinct from the longer half-lives of pERK2 under either condition (Figure S5). Importantly, these data further supported the notion that RSK was not critical to the stability of pERK2 or, therefore, the R45-pERK2 complex.

We previously showed that directed and nearly complete siRNA knockdown of total ERK consistently left residual levels of 30% and 48% of the control levels for pERK1 and pERK2, respectively, which were sufficient to drive lytic gene expression and virus production [1]. Thus, we expected that the modest changes in pERK levels from RSK siRNA knockdown would also have little to no effect on virion production. To test this, we first probed for two lytic proteins, MCP and R45 (Figure 8D). Although the levels of MCP remained unchanged, R45 levels were significantly (approximately 60%) decreased in the siRSK1/2 condition (Figure 8E). Since ORF45 is important for virus production in KSHV ([33], [34], [67]) and RRV (Figure S6), we hypothesized that these changes would negatively affect viral titer (Figure 8F). To our surprise, despite the significant reduction in R45 levels, viral titers did not appreciably decrease, suggesting that the 40% residual levels of R45 was above a minimal threshold necessary to sustain control levels of ERK activation and virus production.

## Discussion

One mechanism viruses use to promote their own propagation within host cells is the manipulation of signaling cascades, particularly the mitogen activated-protein kinase (MAPK) pathways [12]–[14], [20], [28], [68]. We previously reported elevated levels of phosphorylated ERK as late as 48 hours post-infection, though those studies examined the roles of ERK isoforms in RRV infection and virion assembly [1]. In the current study, we focused on the effects of sustained ERK activation as well as the mechanism underlying this activation during de novo RRV infection. We found that pERK levels increase minimally as early as 30 minutes post-infection and then drop by 2.5 h before beginning a continual rise until cell lysis, 48–72 h p.i. Sharma-Walia et al. showed that the addition of soluble KSHV viral envelope glycoprotein B (gB) to cultured fibroblasts or endothelial cells results in a rapid, but transient induction of ERK phosphorylation. However, we observed a similar initial transient increase in pERK in UI as well as UV-RRV infected cells at 30 minutes, suggesting that, in our experimental system, any initial increase in pERK levels due to virus binding is likely masked by the stimulation from the addition of fresh serum-containing media since that step was common among all three experimental conditions and serum can stimulate a rapid and transient induction of ERK phosphorylation [69].

Several viruses, including KSHV, influenza virus, bovine herpesvirus type 1, and adenovirus, that depend on the ERK pathway for virus production display a biphasic activation pattern following infection with the initial phase reflecting virus binding and entry and the later phase coinciding with viral gene expression [13], [25], [26], [32], [70]. Our findings with RRV infection indicate that after an initial transient rise following the change of media, pERK levels in UI and UV-RRV infected cells remain low through 48 h. In marked contrast, infection with intact RRV result in pERK levels rising continuously until the cells lysed (even through 72 h, data not shown). This distinct pattern of continued ERK activation and accumulation during RRV lytic infection suggest that a specific viral gene product(s) might contribute directly or indirectly to this effect throughout lytic infection. Our data implicated that R45 is the most likely candidate (see below).

### Consequences of sustained ERK activation in RRV infection

Though cell signaling events are typically characterized as rapid and transient, several groups have observed both transient and sustained activation of MAP kinases depending on the stimuli and cell type ([71] and reviewed in [47]). For example, exposure of PC12 cells to nerve growth factor (NGF) leads to sustained ERK activation, while addition of epidermal growth factor (EGF) results in transient activation [71]–[73]. Factors such as duration and magnitude of ERK activation can affect the integration of extracellular signals, thereby altering downstream biological responses [74]. In the case of PC12 cells, sustained activation of ERK cause cells to differentiate into sympathetic-like neurons, while transient activation results in proliferation [75]. In contrast, sustained ERK activation in fibroblasts correlates with S phase entry and proliferation [73], [76], [77]. It is unlikely that lytic RRV infection of RhF would lead to a proliferative phenotype due to the simultaneous expression of viral factor(s) that prevent cells from entering S phase [78]–[81].

Another consequence of sustained ERK activation in fibroblasts is nuclear localization of pERK despite its not containing a nuclear localization or export signal [47], [79]. The detailed mechanism of ERK nuclear translocation remained elusive until recent evidence demonstrated that the kinase insert domain in ERK contains a nuclear translocation signal (NTS), allowing it to interact with importin-7 and facilitating its transport through nuclear pore complexes [82]. We detected pERK in both the cytoplasm and nucleus at 24 h p.i. but by 48 h p.i., the majority of pERK was concentrated in the nucleus (Figure 2), leading us to originally hypothesize that substrate activation might be skewed towards nuclear targets at later times p.i. Instead, we found phosphorylation of bothnuclear and cytoplasmic substrates following infection, suggesting persistent ERK activation in both cellular compartments.

### A role for R45 in sustained ERK activation during RRV infection

Several studies have demonstrated that the expression of specific lytic KSHV genes leads to ERK phosphorylation, including the lytic gene products, vGPCR (ORF74) and ORF45 (K45) [24], [30], [83], [84]. The elegant work from Zhu's laboratory showed that K45 interacts with p90RSK and ERK to nucleate the formation of phosphatase-resistant complexes [34]. In those complexes, K45 protects both pERK and pRSK from phosphatases, leading to elevated levels of their phosphorylated forms. We reasoned R45 could play a similar role in sustaining ERK activation in RRV infection since ORF45 homologs exist in RRV and other gammaherpesviruses, including EBV. Nevertheless, significant sequence differences exist between R45 and K45, suggesting the formal possibility that functional differences might also exist between the two homologs. R45, for example, is 353 residues, while K45 is 407aa and although these proteins are most similar in the N (49% identical) and C (55% identical) termini, they are divergent in their central region [85]. Despite these differences, our data stemming from co-localization (confocal and FRET analyses) (Figures 5 and 7), co-immunoprecipitation (Figure 4) as well as both gain and loss (Figure 3 and Figure S3) of R45 function experiments, together, argue that R45 plays a major role in the marked elevations in pERK2 with which it forms stable complexes (Figures 4 and 6). Further, our data suggest that a) R45 sequesters and protect the phosphorylated state of ERK2 without hindering the latter's ability to activate its nuclear targets and b) sufficient amounts of uncomplexed pERK remain in the cytoplasm to support activation of its cytoplasmic substrates (Figure 2B). Interestingly, we noted that although the activated forms of the ERK targets, MNK, RSK, and Elk-1 rose, their total protein levels were lower in infected compared to UI cells. We reasoned this latter phenomenon could be due to the expression of the gammaherpesvirus protein, SOX (of which there are homologs in other herpesviruses [59], [86]) that promotes the shut off of host cell gene expression during lytic replication [60].

### R45 preferentially protects pERK2 over pERK1

In our previous work, we described distinct roles, for ERK1 and ERK2 in RRV structure and production [1]. We demonstrated that ERK1 is a negative regulator of virus production and ERK2 is preferentially activated (and subsequently incorporated into infectious virions) during infection. In light of these data, we measured the levels of both pERK1 and pERK2 after R45 transfection. We hypothesized that R45 expression would lead to increased pERK2 levels. As expected, upon R45 transfection alone, the levels of pERK2, but not pERK1, were statistically greater. Together, these data, along with the marked differences in the stability of pERK2 over pERK1, as we have discussed above, suggest that R45 plays a role in the preferential activation of ERK2 during RRV infection, which may be important for sustaining lytic gene expression. We discuss this point further below.

### A nonessential role for RSK

The model for ERK activation in KSHV involves the direct interaction between K45 and RSK. Zhu et al. proposed that in lytic replication, pERK binds to and activates RSK, which in turn interacts with K45 [24]. In this complex, pERK and pRSK remain protected from dephosphorylation. Although the RSK binding domain mapped in K45 is only 49% identical to the same region in R45, we observed an augmentation of pERK levels when we co-expressed RSK2 with R45 but only in the setting of ectopic overexpression of both proteins. In contrast, during bona fide infection, we found that siRNA knockdown of RSK showed only a minimal effect on pERK accumulation (Figure 8A and C) and titers of released virus were essentially unchanged (Figure 8F). Thus, although the heterotrimeric complexes that exist in KSHV also formed following de novo RRV infection, RSK's role in the ongoing accumulation of nuclear pERK2 is perhaps relatively less important than during KSHV infection. On the other hand, we also recognize that RSK knockdown in our experiments was incomplete, leaving residual pools of RSK (Figure 8B). It is possible that this amount (approximately 20% of control levels) could be sufficient to support the formation of ORF45-pERK2-pRSK complexes and, in turn, viral protein translation and virion production, thereby explaining the unchanged titers with RSK knockdown that we observed. We earlier found that residual levels of pERK were sufficient to support robust virion production while complete pharmacologic inhibition of the MEK-ERK pathway prevented it [1].

Though our data are consistent with the KSHV-based model and suggest that R45 forms complexes that protect pERK2 from dephosphorylation, we also reasoned that since activation of the MEK/ERK pathway is essential during lytic RRV infection, other viral and cellular factors might also contribute to this process. For example, RRV infection could potentially suppress the expression of ERK-specific phosphatases such as MKP-1 and 4, leading to increased levels of pERK. Of note, we probed for MKP-1 at 24 and 48 h p.i. and found no change in its levels following infection compared to uninfected controls (data not shown). These data suggest that at least for this one ERK-specific phosphatase, down regulation of its expression does not appear to play a contributory role in sustained activation during RRV infection, though further experiments are necessary to draw this conclusion definitively. Similarly, RRV infection could, in addition, greatly stimulate MEK activity leading to the marked increases in pERK accumulation. However, we found that pMEK levels increased only minimally (<1.4 fold) in RRV infected cells compared to uninfected controls and, thus, might contribute to but are unlikely to be the major driving force the elevations in pERK (Figure S7).

Though we were able to detect co-localization of pERK with both R45 and RSK in the nuclei of infected cells, the fluorescence microscopy also indicated that it was also present separately within the cytoplasm (Figures 5B and 7A). In our experiments using the MEK inhibitor, U0126 to assess the stability of the complex, we found that pERK2, but not pERK1, was protected from phosphatases within the complex as pERK2 levels declined slowly, consistent with a slow R45 off rate (Figure 6). In marked contrast, pERK1 levels declined rapidly, consistent with its being freely accessible to (i.e. unprotected from) the cell's phosphatases. pERK2's ability to remain phosphorylated and to phosphorylate downstream targets suggests that R45 may be acting as an allosteric inhibitor of dual-specificity phosphatases such as MKPs [18], [61], [62]. The kinase interacting motifis (KIM) of these phosphatases interact with common docking (CD) sites on MAPK (i.e. pERK2), but these sites are outside of the kinase domain [62]. Therefore, R45 may bind to the CD motif on pERK2, preventing it from interacting with MKPs, while not interfering with its ability it to interact with both its upstream activators and downstream targets.

In summary, this is the first study of which we are aware to examine the localization of activated ERK at late times p.i., and the activation of ERK substrates in response to gammaherpesvirus infection. We have demonstrated that ERK activation is sustained throughout the course of RRV infection and the accumulation of pERK activates both nuclear and cellular targets. We note, however, that our data do not formally distinguish whether the complexed or only the free pERK is able to interact with and activate these substrates. Additionally, we presented evidence that ORF45's role in ERK activation, through the formation of complexes with RSK and ERK, is conserved in primate gammaherpesviruses, though the role for RSK seems less essential in RRV than in KSHV infection. Finally, we demonstrated that R45 selectively protects pERK2, the dominant ERK isoform also found within the tegument of RRV, further suggesting a role for R45 in pERK2 intravirion incorporation. These data, along with our earlier findings [1], emphasize how gammaherpesviruses have evolved to induce persistent accumulation of pERK to drive lytic gene expression and virion production.

## Supporting Information

Figure S1 **Activated ERK localizes to the nucleus upon non-viral stimulation.** Standard immunofluorescence images of unstimulated (top panels) and TPA-stimulated (15 min) RhF (bottom panels). Cells were stained for DAPI (blue; 1^st^ column) and pERK (red; 2^nd^ column). Images were merged in the 3^rd^ column. Magnification 40×.
(TIFF)

Figure S2 **Subcellular localization of ERK substrates 48 h post RRV infection.** Confocal immunofluorescence images of RhF either uninfected (UI) or 48 h p.i. (RRV) stained for pRSK, total MNK1 and pELK-1. All cells were also stained with DAPI and images were merged to show both the nuclei and the fluorescent signal from each indicated protein. Magnification 63×.
(TIFF)

Figure S3 **During RRV infection, the rise in intracellular pERK levels correlates with the kinetics of R45 expression and is inhibited by R45 knock down.** (**A**) Quantitative immunoblots of RRV-infected RhF (MOI of 10) at increasing times p.i. 10 ug of cell lysate (10–15% of total lysate) were loaded per lane. Blots were probed with the following antibodies: anti-R45 and anti-Ran (loading control for cellular extracts). (**B**) Quantitative immunoblot of UI (lane 1) and infected (lanes 2 and 3) RhF. 24 h prior to infection, RhF were transfected with siCNL (lane 2) or siR45 (lane 3). Cells were harvested 48 h p.i. and 10 ug whole cell lysate (∼10–15% of total lysate) were probed for the proteins indicated to the right. (**C**) Graphic representation of R45 and pERK2 levels from 6 independent experiments described in (B). Data represent the mean +/− SEM. As compared to siCNL, R45 and pERK2 levels were statistically different in the siR45 conditions (p values were 0.0001 and 0.0001 respectively).
(TIFF)

Figure S4 **U0124, an inactive analog of the MEK inhibitor, U0126, does not inhibit ERK activation during RRV infection.** Quantitative immunoblot of uninfected (UI; lane 1) and infected (lanes 2–6) RhF treated with DMSO (lane 2), 10 or 50 uM U0126 (lanes 3 and 4, respectively) or 10 or 50 uM U0124 (lanes 5 and 6). Cells were harvested and 10 ug of lysate (∼10–15% of total whole cell lysate) from each condition were probed for pERK as well as Ran to control for loading.
(TIFF)

Figure S5 **Delayed decay of pERK2 during RRV infection is independent of RSK expression.** (A) RhF were transfected with siCNL or siRSK1+2 (siRSK) and 24 h later infected with RRV at an MOI of 2.5. 48 h p.i. cultures were treated with DMSO (top) or the MEK inhibitor, U0126 (bottom) for up to 10 h. 10 ug of cell lysate (10–15% of total lysate) were loaded per lane and immunoblotted with antibodies to pERK, RSK1+2, and GAPDH to control for loading. (B) Graphical representation of two independent experiments described in (A) with levels of pERK1 and pERK2 at each time point first normalized to GAPDH and then expressed as the ratio of their values under U0126 or DMSO conditions. We set the ratios for pERK1 and for pERK2 levels to 1.0 for the siCNL samples at the zero hour time point. Subsequent time points are relative to these initial values. Dashed lines indicate the samples that received siCNL and solid lines siRSK. Data are the mean from the two experiments with error bars representing the range. R^2^ values indicate the coefficient of determination for each curve and the p-values indicate the level of significance difference using the extra sum-of-squares F test (Prism 6.0 d software) between the decay curves of each pERK1 and pERK2 with (siRSK) or without (siCNL) RSK knockdown (p values were 0.0029 and 0.799 respectively). (Of note, as with Figure 6, the lower MOI of 2.5 helped minimize the degree of lysis between p.i. hours 48 to 58).
(TIFF)

Figure S6 **Knock down of R45 greatly reduces the production of infectious virions.** Viral titers from the supernatants of RRV infected RhF pre-treated with either control (siCNL) or R45-directed (siR45) siRNA. Each column is representative of mean from 3 independent experiments +/−SEM. As compared to siCNL, viral titers were significantly reduced in the siR45 condition (p value was 0.01).
(TIFF)

Figure S7 **RRV infection leads to marked increase in activated ERK with only a minimal increase in MEK activation.** Quantitative immunoblot of uninfected (UI) and infected (Inf; MOI of 5) RhF at 48 h p.i. 10 ug cell lysate (∼10–15% total whole cell lysate) was probed for the proteins indicated to the right of the figure.
(TIFF)

Figure S8 **Full immunoblot used for** **Figure 3A:** **RRV ORF45 (R45) expression leads to the phosphorylation of ERK.** Dotted lines indicate the lanes digitally juxtaposed for Figure 3A.
(TIFF)

Figure S9 **Full immunoblot used for** **Figure 8A:** **RSK is not required for ERK activation.** Dotted lines indicate the lanes digitally juxtaposed for Figure 3A.
(TIFF)

Table S1 **Summary of PFRET data analysis.** Donor to acceptor ratios (D/A ratio), FRET efficiency (E%), and distance between fluorophores (r, in nm) were calculated for each region of interest (ROIs). On average, over 300 ROIs were analyzed in each experiment. The data represent the mean of two independent experiments for each condition. For r (nm), values represent the mean +/− the SEM.
(XLSX)
