# Correction: Progressive Accumulation of Activated ERK2 within Highly Stable ORF45-Containing Nuclear Complexes Promotes Lytic Gammaherpesvirus Infection

**DOI:** 10.1371/journal.ppat.1004147

**Published:** 2014-04-28

**Authors:** The PLOS Pathogens Staff

Some of the author contributions for Melissa S. Anderson were omitted. The following are the correct author contributions for this paper:

Conceived and designed the experiments: ENW DHK. Performed the experiments: ENW MSA MSL. Analyzed the data: ENW MSA DHK. Contributed reagents/materials/analysis tools: MSA. Wrote the paper: ENW DHK
