# Supplementary figures and images for: Progressive Accumulation of Activated ERK2 within Highly Stable ORF45-Containing Nuclear Complexes Promotes Lytic Gammaherpesvirus Infection
Source: PLoS Pathog. 2014 Apr 10;10(4):e1004066. doi: 10.1371/journal.ppat.1004066 (PMC3983062; doi:10.1371/journal.ppat.1004066)

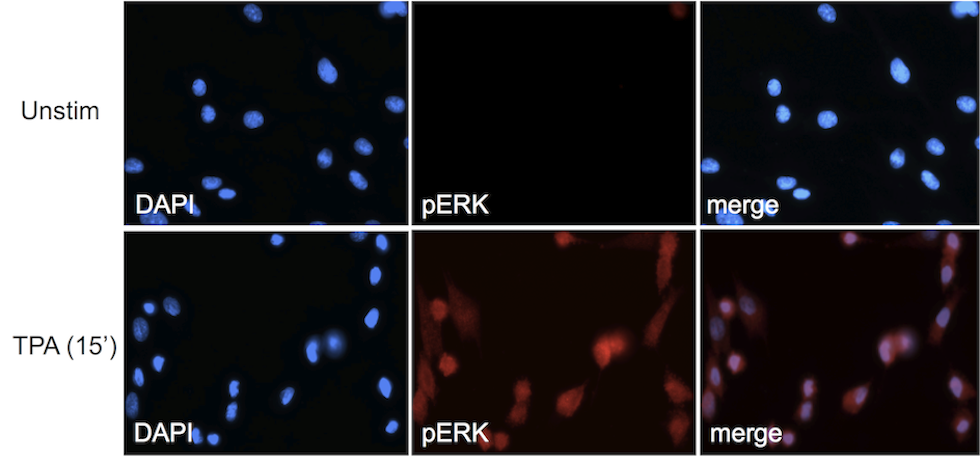

Supplement: Figure S1 — Activated ERK localizes to the nucleus upon non-viral stimulation. Standard immunofluorescence images of unstimulated (top panels) and TPA-stimulated (15 min) RhF (bottom panels). Cells were stained for DAPI (blue; 1st column) and pERK (red; 2nd column). Images were merged in the 3rd column. Magnification 40×. (TIFF) [file ppat.1004066.s001.tiff]

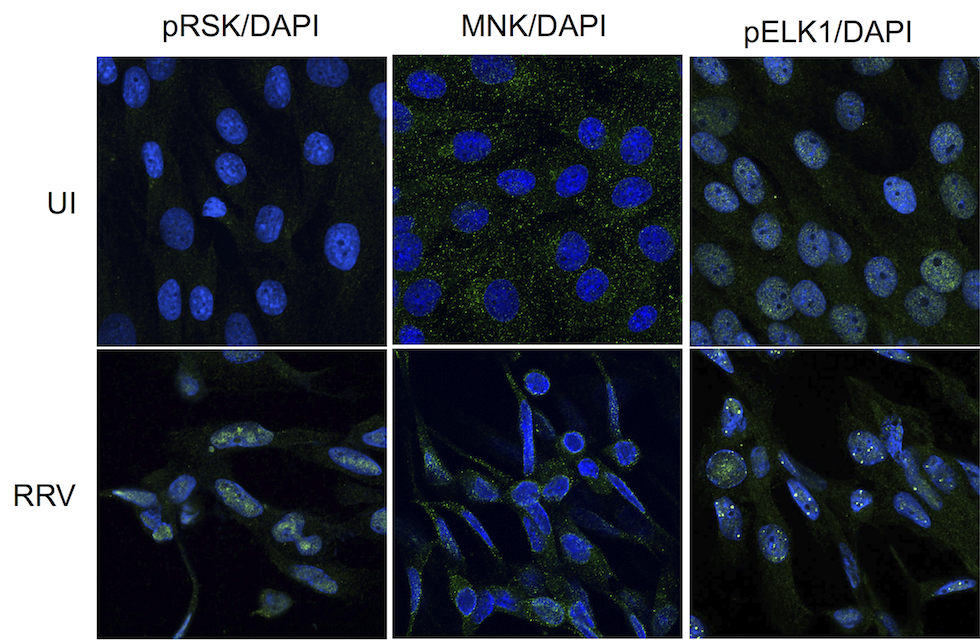

Supplement: Figure S2 — Subcellular localization of ERK substrates 48 h post RRV infection. Confocal immunofluorescence images of RhF either uninfected (UI) or 48 h p.i. (RRV) stained for pRSK, total MNK1 and pELK-1. All cells were also stained with DAPI and images were merged to show both the nuclei and the fluorescent signal from each indicated protein. Magnification 63×. (TIFF) [file ppat.1004066.s002.tiff]

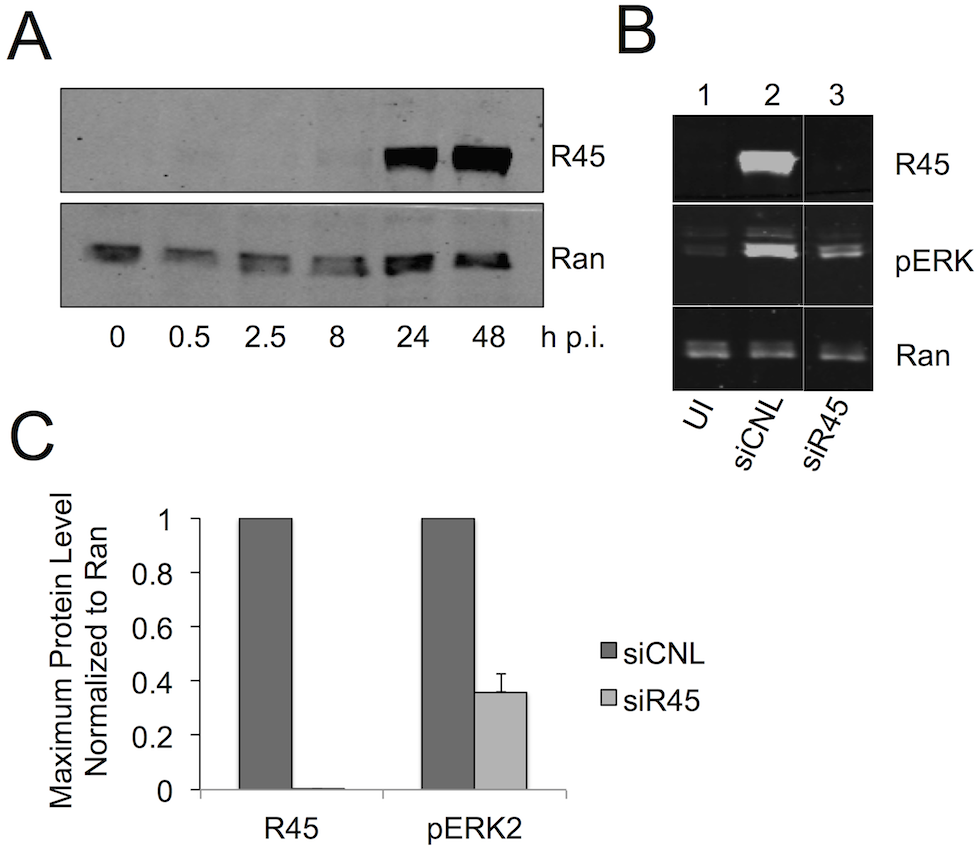

Supplement: Figure S3 — During RRV infection, the rise in intracellular pERK levels correlates with the kinetics of R45 expression and is inhibited by R45 knock down. (A) Quantitative immunoblots of RRV-infected RhF (MOI of 10) at increasing times p.i. 10 ug of cell lysate (10–15% of total lysate) were loaded per lane. Blots were probed with the following antibodies: anti-R45 and anti-Ran (loading control for cellular extracts). (B) Quantitative immunoblot of UI (lane 1) and infected (lanes 2 and 3) RhF. 24 h prior to infection, RhF were transfected with siCNL (lane 2) or siR45 (lane 3). Cells were harvested 48 h p.i. and 10 ug whole cell lysate (∼10–15% of total lysate) were probed for the proteins indicated to the right. (C) Graphic representation of R45 and pERK2 levels from 6 independent experiments described in (B). Data represent the mean +/− SEM. As compared to siCNL, R45 and pERK2 levels were statistically different in the siR45 conditions (p values were 0.0001 and 0.0001 respectively). (TIFF) [file ppat.1004066.s003.tiff]

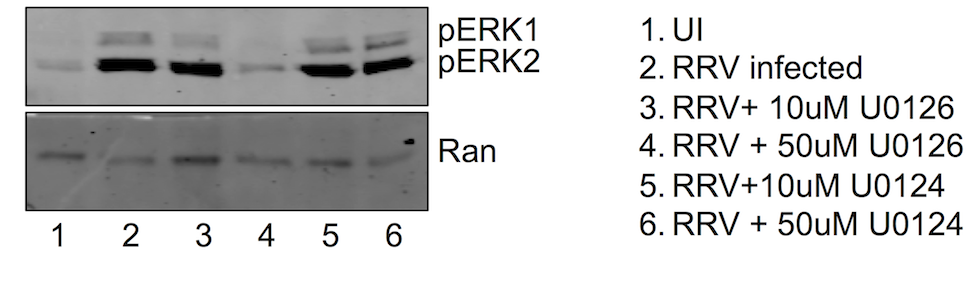

Supplement: Figure S4 — U0124, an inactive analog of the MEK inhibitor, U0126, does not inhibit ERK activation during RRV infection. Quantitative immunoblot of uninfected (UI; lane 1) and infected (lanes 2–6) RhF treated with DMSO (lane 2), 10 or 50 uM U0126 (lanes 3 and 4, respectively) or 10 or 50 uM U0124 (lanes 5 and 6). Cells were harvested and 10 ug of lysate (∼10–15% of total whole cell lysate) from each condition were probed for pERK as well as Ran to control for loading. (TIFF) [file ppat.1004066.s004.tiff]

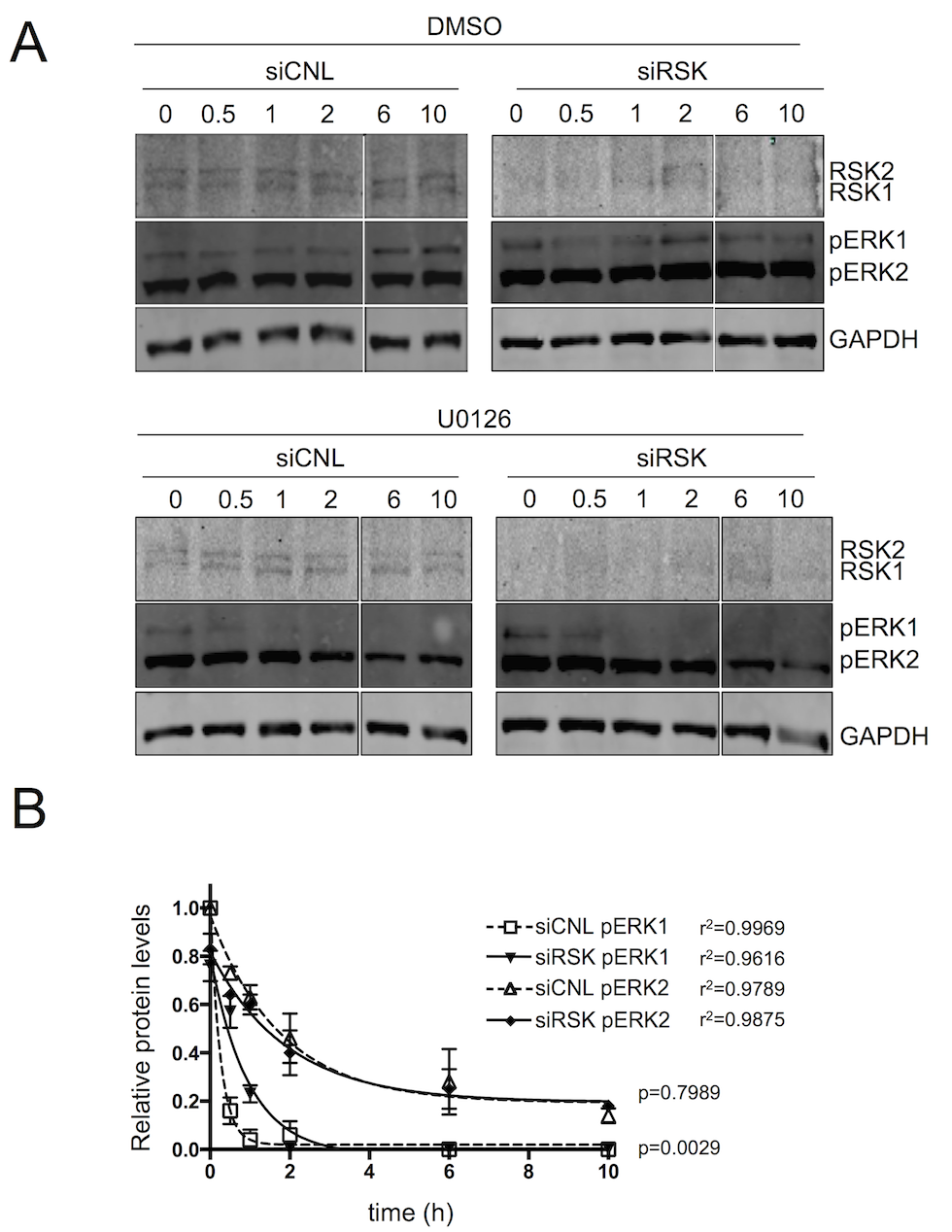

Supplement: Figure S5 — Delayed decay of pERK2 during RRV infection is independent of RSK expression. (A) RhF were transfected with siCNL or siRSK1+2 (siRSK) and 24 h later infected with RRV at an MOI of 2.5. 48 h p.i. cultures were treated with DMSO (top) or the MEK inhibitor, U0126 (bottom) for up to 10 h. 10 ug of cell lysate (10–15% of total lysate) were loaded per lane and immunoblotted with antibodies to pERK, RSK1+2, and GAPDH to control for loading. (B) Graphical representation of two independent experiments described in (A) with levels of pERK1 and pERK2 at each time point first normalized to GAPDH and then expressed as the ratio of their values under U0126 or DMSO conditions. We set the ratios for pERK1 and for pERK2 levels to 1.0 for the siCNL samples at the zero hour time point. Subsequent time points are relative to these initial values. Dashed lines indicate the samples that received siCNL and solid lines siRSK. Data are the mean from the two experiments with error bars representing the range. R2 values indicate the coefficient of determination for each curve and the p-values indicate the level of significance difference using the extra sum-of-squares F test (Prism 6.0 d software) between the decay curves of each pERK1 and pERK2 with (siRSK) or without (siCNL) RSK knockdown (p values were 0.0029 and 0.799 respectively). (Of note, as with Figure 6, the lower MOI of 2.5 helped minimize the degree of lysis between p.i. hours 48 to 58). (TIFF) [file ppat.1004066.s005.tiff]

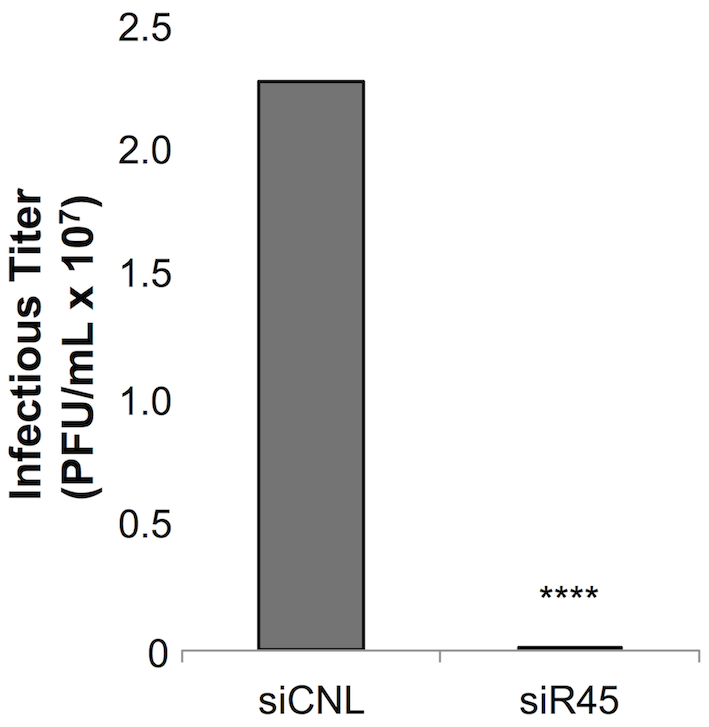

Supplement: Figure S6 — Knock down of R45 greatly reduces the production of infectious virions. Viral titers from the supernatants of RRV infected RhF pre-treated with either control (siCNL) or R45-directed (siR45) siRNA. Each column is representative of mean from 3 independent experiments +/−SEM. As compared to siCNL, viral titers were significantly reduced in the siR45 condition (p value was 0.01). (TIFF) [file ppat.1004066.s006.tiff]

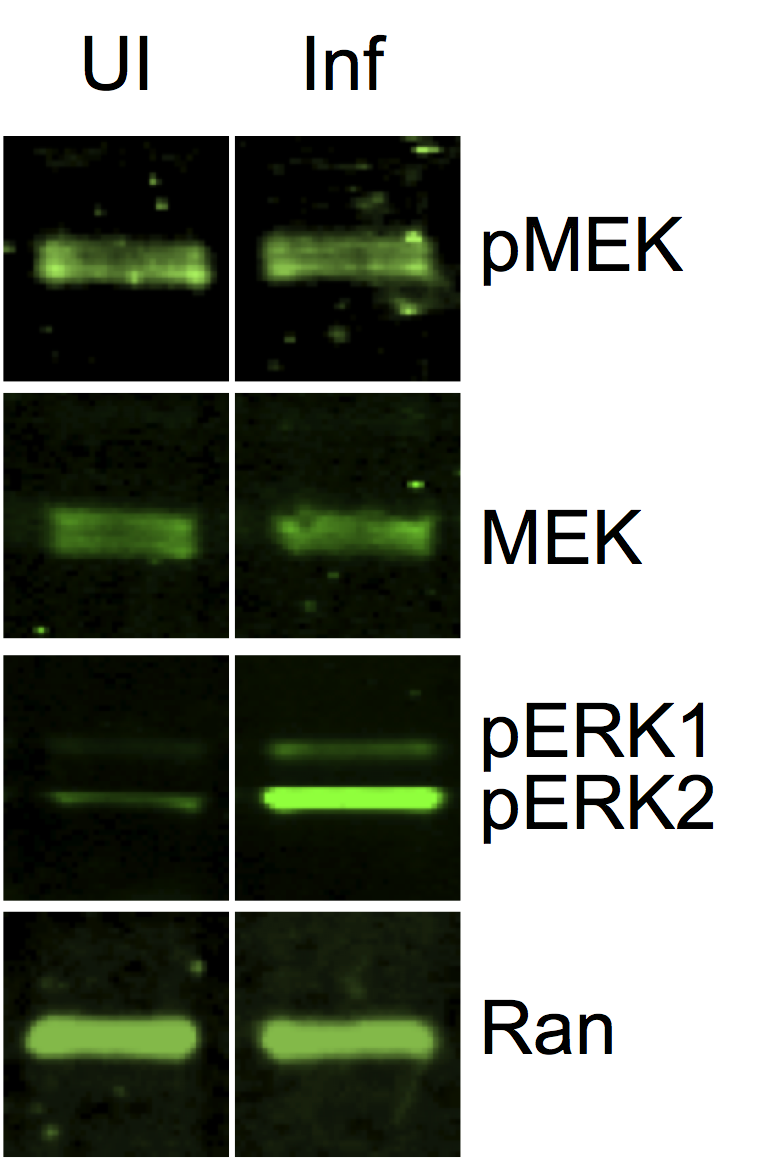

Supplement: Figure S7 — RRV infection leads to marked increase in activated ERK with only a minimal increase in MEK activation. Quantitative immunoblot of uninfected (UI) and infected (Inf; MOI of 5) RhF at 48 h p.i. 10 ug cell lysate (∼10–15% total whole cell lysate) was probed for the proteins indicated to the right of the figure. (TIFF) [file ppat.1004066.s007.tiff]

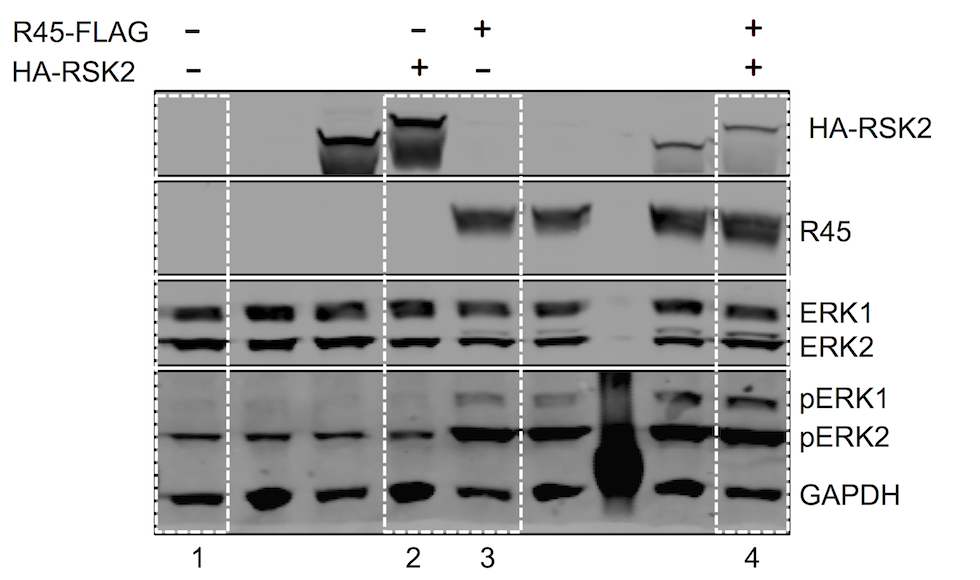

Supplement: Figure S8 — Full immunoblot used for Figure 3A: RRV ORF45 (R45) expression leads to the phosphorylation of ERK. Dotted lines indicate the lanes digitally juxtaposed for Figure 3A. (TIFF) [file ppat.1004066.s008.tiff]

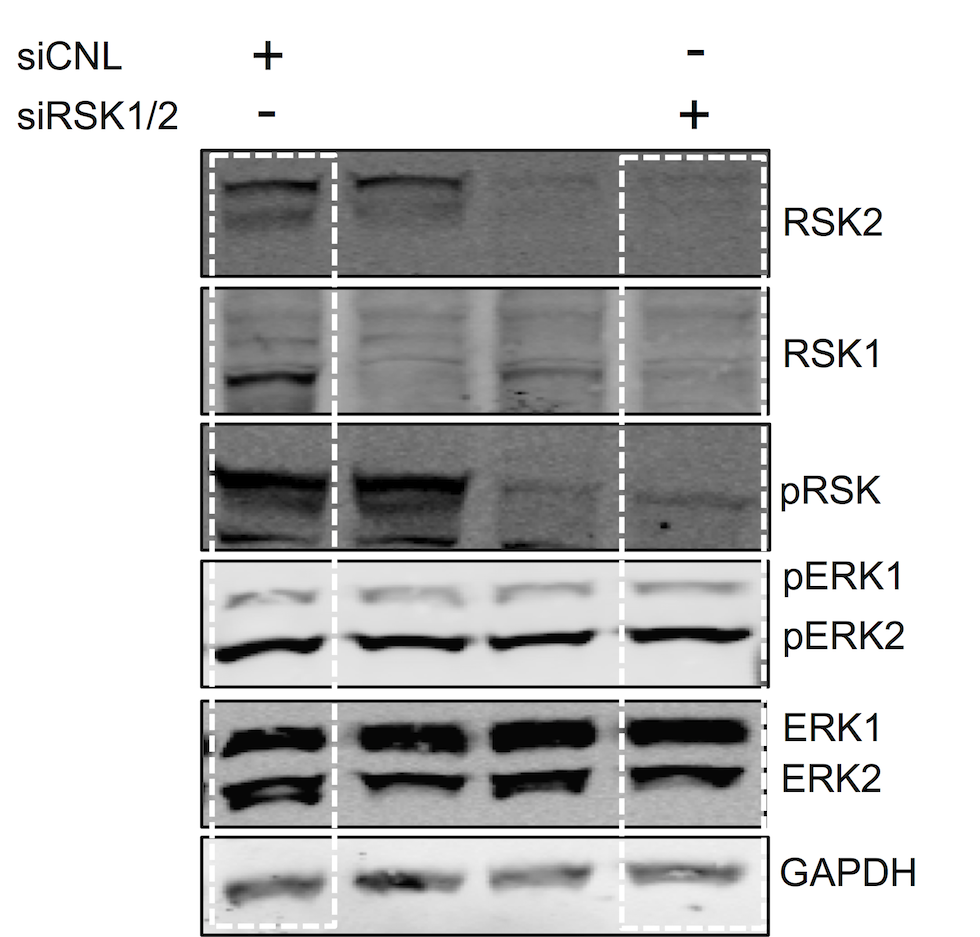

Supplement: Figure S9 — Full immunoblot used for Figure 8A: RSK is not required for ERK activation. Dotted lines indicate the lanes digitally juxtaposed for Figure 3A. (TIFF) [file ppat.1004066.s009.tiff]
